# Bile acids promote the caveolae-associated entry of swine acute diarrhea syndrome coronavirus in porcine intestinal enteroids

**DOI:** 10.1371/journal.ppat.1010620

**Published:** 2022-06-13

**Authors:** Qi-Yue Yang, Yong-Le Yang, Yi-Xin Tang, Pan Qin, Gan Wang, Jin-Yan Xie, Shu-Xian Chen, Chan Ding, Yao-Wei Huang, Shu Jeffrey Zhu

**Affiliations:** 1 Key Laboratory of Animal Virology of Ministry of Agriculture, Center for Veterinary Sciences, Zhejiang University, Hangzhou, People’s Republic of China; 2 Department of Veterinary Medicine, Zhejiang University, Hangzhou, People’s Republic of China; 3 Shanghai Veterinary Research Institute, Chinese Academy of Agricultural Sciences, Shanghai, People’s Republic of China; 4 Jiangsu Co-innovation Center for Prevention and Control of Important Animal Infectious Diseases and Zoonoses, Yangzhou, People’s Republic of China; 5 Guangdong Laboratory for Lingnan Modern Agriculture, Guangzhou, People’s Republic of China; 6 Department of Critical Care Medicine, Sir Run Run Shaw Hospital, Zhejiang University School of Medicine, Hangzhou, People’s Republic of China; University of Michigan, USA, UNITED STATES

## Abstract

Intestinal microbial metabolites have been increasingly recognized as important regulators of enteric viral infection. However, very little information is available about which specific microbiota-derived metabolites are crucial for swine enteric coronavirus (SECoV) infection *in vivo*. Using swine acute diarrhea syndrome (SADS)-CoV as a model, we were able to identify a greatly altered bile acid (BA) profile in the small intestine of infected piglets by untargeted metabolomic analysis. Using a newly established ex vivo model–the stem cell-derived porcine intestinal enteroid (PIE) culture–we demonstrated that certain BAs, cholic acid (CA) in particular, enhance SADS-CoV replication by acting on PIEs at the early phase of infection. We ruled out the possibility that CA exerts an augmenting effect on viral replication through classic farnesoid X receptor or Takeda G protein-coupled receptor 5 signaling, innate immune suppression or viral attachment. BA induced multiple cellular responses including rapid changes in caveolae-mediated endocytosis, endosomal acidification and dynamics of the endosomal/lysosomal system that are critical for SADS-CoV replication. Thus, our findings shed light on how SECoVs exploit microbiome-derived metabolite BAs to swiftly establish viral infection and accelerate replication within the intestinal microenvironment.

## Author summary

Bile acids (BAs), a commonly studied category of microbial metabolites, have long been acknowledged to have proviral or antiviral activities. Recent studies using different swine enteric coronaviruses (SECoVs) showed that BA play an important role in regulating viral replication *in vitro*. A mechanistic understanding of how BA regulates SECoV replication in small intestinal enterocytes is lacking. Herein, we utilized an emerging highly pathogenic SECoV, swine acute diarrhea syndrome (SADS)-CoV, which possesses the potential for zoonotic transmission, to investigate the crucial role of BA in modulating viral replication in porcine intestinal enteroids (PIEs). Our observations explain how BAs acts on epithelial cells to enhance SADS-CoV replication by inducing caveolae-mediated endocytosis and endosomal acidification, altering the dynamics of viral trafficking through the cellular endosomal/lysosomal system. Our results shed light on the role of BAs in the rapid establishment of SECoV infection within the intestinal microenvironment.

## Introduction

The mammalian gastrointestinal (GI) tract harbors an enormously diverse microbial community (termed ‘microbiota’) that develops a mutualistic relationship with its host, forming a complex ecosystem over millions of years of coevolution [1]. The intestinal microbiome generates immensely disparate metabolic products that can modulate host physiological activity and immune responses directly or indirectly [2]. Thus, infection by enteric viruses is not just a simple biological event between pathogen and host target cell, but rather a complicated process that takes place in the context of the intestinal microenvironment.

Cumulative evidence supports the view that microbial metabolites can regulate enteric viral infection [3–5]. Among them, bile acids (BAs) have been shown to play crucial roles in enhancing replication of enteric viruses such as porcine sapoviruses (PoSaV), porcine enteric calicivirus (PEC) and noroviruses (NoVs) [6–8]. The replication of PEC in the porcine kidney cell line LLC-PK1 is dependent on the presence of BAs in the culture medium for at least two reasons: 1) BAs facilitate PEC escape from the endosome into cytoplasm for initiation of viral replication [8]; 2) BAs inhibit cellular innate immunity by downregulating phosphorylation of signal transducer and activator of transcription 1 (STAT1) upon PEC infection in LLC-PK1 [9]. Human NoV (HuNoV) subtype GII.3 replication in human intestinal enteroids (HIE) depends on the enhanced endosomal/lysosomal acidification and activation of sphinogomyelinase ASM caused by BAs [10,11]. The major capsid protein (VP1) of murine norovirus (MNV) binds to BAs, triggering a structural variation in the virion that enhances receptor binding and viral infectivity, as well as blocking antibody neutralization [12,13].

Very few studies have focused on how BAs influence replication of swine enteric coronaviruses (SECoVs), though it was recently demonstrated that BAs increased infectivity of porcine epidemic diarrhea coronavirus (PEDV) strain icPC22A in Vero cells and the porcine small intestinal epithelial cell line IPEC-DQ [14]. However, a later study reported that BAs had antiviral activity against another SECoV, porcine deltacoronavirus (PDCoV), reducing its replication in LLC-PK1 and IPEC-J2 cells [15]. These seemingly contradictory outcomes suggest that BAs may modulate replication of distinct SECoVs very differently. Unfortunately, a mechanistic understanding of how BA regulates SECoVs replication in small intestinal enterocytes is still lacking.

A novel emerging pathogenic SECoV, swine acute diarrhea syndrome (SADS)-CoV, was first reported in suckling piglets with severe diarrhea in Guangdong, China in 2017 [16,17]. SADS-CoV preferentially infects the GI tract and causes clinical symptoms including acute vomiting and watery diarrhea [16,18]. This novel alphacoronavirus is most closely related to bat coronavirus HKU2 [16,19], and it is capable of infecting cell lines from several species including pigs, nonhuman primates and humans, raising the concern that it might possess the potential to jump to human beings [20–22]. The typical clinical presentation in the GI tract makes SADS-CoV a perfect model for the study of the critical role of BA in the regulation of SECoV replication.

Primary BAs including cholic acid (CA) and chenodeoxycholic acid (CDCA) are synthesized from cholesterol-derived precursor molecules, conjugated to either taurine (mainly in rodents) or glycine (primarily in humans) within hepatocytes and excreted into the small intestines, where commensal bacteria deconjugate and convert them into secondary BAs, such as deoxycholic acid (DCA) and lithocholic acid (LCA) [23]. Approximately 95% of BAs are reabsorbed in the distal ileum and transported back to the liver to complete enterohepatic circulation. This makes the ileum the site of a large pool of various BAs with relatively high concentrations [24]. Thus, we hypothesized that SADS-CoV might take advantage of this BA-rich microenvironment to swiftly establish early infection and facilitate its spread in small intestinal epithelial cells.

To confirm this hypothesis, we used untargeted metabolomics to profile small intestinal metabolites in SADS-CoV-infected piglets vs mock controls, discovering a series of BAs that were greatly enriched. Using porcine intestinal enteroids (PIEs) to mimic intestinal biology and physiology *in vivo*, we modeled the impact of BAs on SADS-CoV replication in intestinal enterocytes. We found that CA induces cellular changes that are of vital importance for SADS-CoV replication, including enhanced caveolae-mediated endocytosis (CavME), endosomal acidification and altered dynamics of the endo-lysosomal system. This novel role of BAs in promoting SECoV replication brings a new perspective on the establishment of viral infection in the intestinal microenvironment *in vivo*.

## Results

### SADS-CoV oral infection leads to a significant increase of BAs in the small intestine of piglets

**Fig 1:**
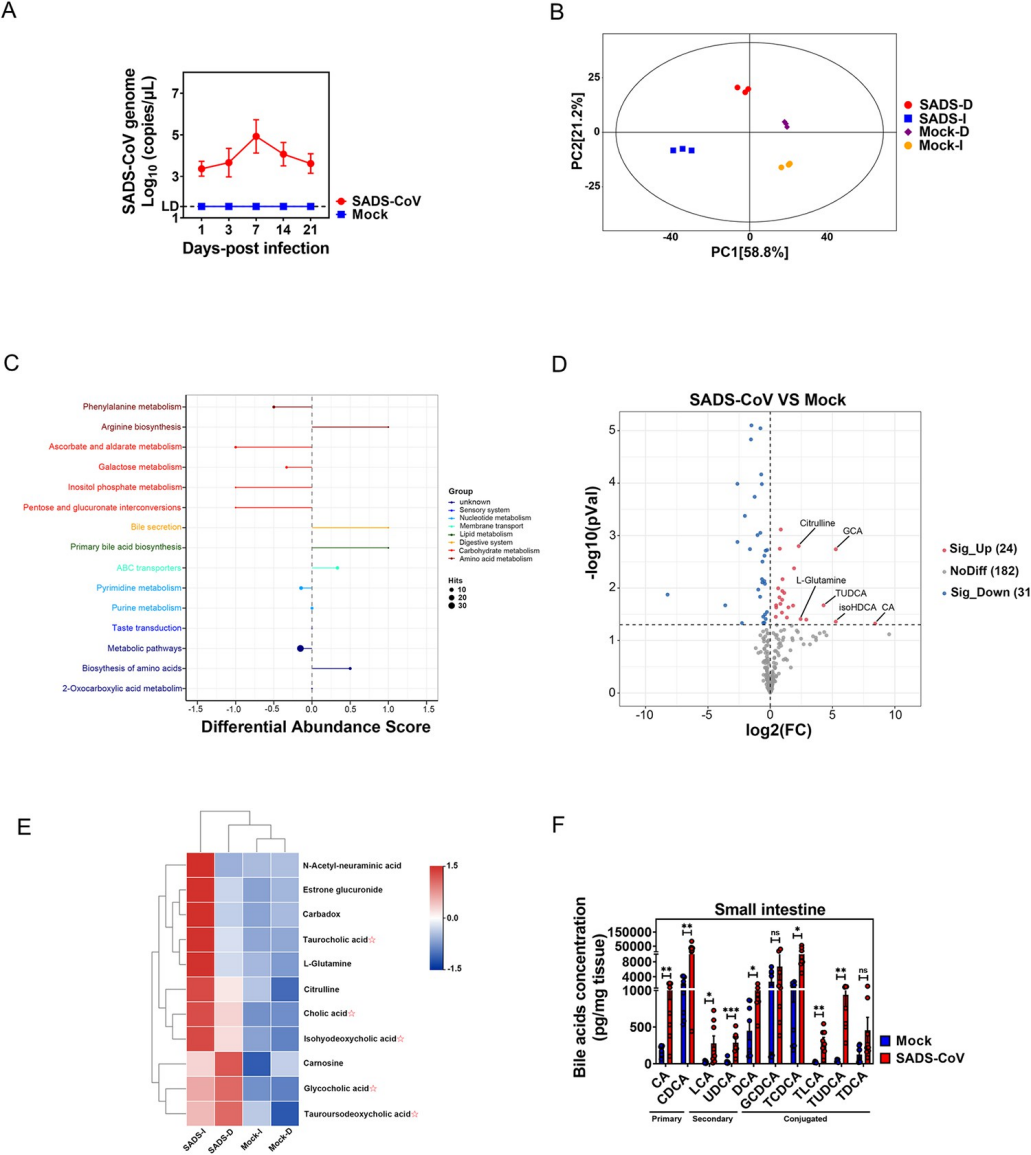
SADS-CoV oral infection leads to a significant increase of bile acids in the small intestine of piglets. (A) Kinetic of viral shedding in fecal swabs from sucking piglets orally infected with SADS-CoV (n = 6). (B) Principal components analysis (PCA) of the duodenum and ileum from SADS-CoV-infected and mock-infected piglets. (C) KEGG analysis of small intestinal metabolites in SADS-CoV-infected and mock-infected piglets. (D) Volcano plot of small intestinal metabolites in small intestine of SADS-CoV-infected and mock-infected animals. (E) Heatmap of small intestinal metabolites in the duodenum and ileum. (F) Small intestinal bile acid (BA) concentrations of SADS-CoV-infected and mock-infected piglets. *P* values were determined by unpaired two-tailed Student’s t test. *: *p* < .05; **: *p* < .01; ***: *p* < .001; ns, not significant.

High viral titers and severe histopathological changes including diminishing capillaries and villous atrophy in the small intestines indicate that SADS-CoV infection is highly efficient and pathogenic in newborn suckling piglets [16,18,25]. Although previous studies did not profile the infection-related metabolites in the small intestine, we speculated that infection may result in perturbations to the gut microenvironment (i.e., redistribution of specific microbiota-derived metabolites) that would enhance SADS-CoV replication. Groups of 3-day-old SPF suckling piglets (n = 6) were orally inoculated with either vehicle or 3×10_5_ PFU of SADS-CoV and monitored for viral shedding. At 7 days post-infection (dpi), coinciding with the peak of viral shedding (Fig 1A), animals were sacrificed, and proximal and distal segments of the small intestine were dissected and subjected to untargeted metabolomics analysis by liquid chromatography-mass spectrometry (LC-MS). As expected, the principal components analysis (PCA) revealed a distinct metabolomic profile in infected piglets from that of the negative controls (Fig 1B). Hierarchical clustering and KEGG analyses suggested that the increased differential metabolites in the small intestines of infected piglets were mainly enriched in lipid metabolism, more specifically primary bile acid biosynthesis (Fig 1C). Indeed, from a total of 364 metabolites that were remarkably upregulated (|log2 FC| >0.5, adjusted *p* < .05) in the small intestines of infected piglets, we discovered primary BAs such as CA, glycocholic acid (GCA), and secondary BAs like isohyodeoxycholic acid (isoHDCA) and tauroursodeoxycholic acid (TUDCA) (Fig 1D). We further compared the differentiated metabolites between the proximal and distal small intestine and discovered that SADS-CoV infection induced significantly higher level of CA, taurocholic acid (TCA) and isoHDCA in the distal, and to a lesser extent in the proximal small intestine (Fig 1E). On the contrary, the increase in GCA and TUDCA was less pronounced in the distal than proximal small intestine (Fig 1E). This finding suggests that SADS-CoV infection induces augmentation of BAs along the small intestine in a tissue-specific manner. To further confirm these results, a targeted metabolic profiling study was performed on BAs, finding a markedly higher absolute concentration of a series of BAs in the small intestine of infected compared to mock-infected piglets (Fig 1F). Collectively, the results from both metabolic profile analyses demonstrated that SADS-CoV infection greatly increased the concentration of BAs in the small intestine, which might be positively correlated with efficient viral replication and epithelial tissue damage.

### PIEs effectively support growth of SADS-CoV

With the advantage of recapitulating the structural and functional features of natural intestinal epithelium *in vivo*, PIEs are useful as a novel *ex vivo* system to study the infections of SECoVs such as PEDV and PDCoV [26,27]. Therefore, we first generated three-dimensional (3D) enteroids from crypts harvested from the duodenum, jejunum or ileum of piglets and dissociate them into 2D enteroids monolayers (S1A and S1B Fig). As shown in S1C Fig, the 2D PIE cultures were stained positive with multiple cellular markers including villin (enterocytes), Ki-67 (proliferating cells), E-cadherin (epithelial tight junction) and chromogranin A (enteroendocrine cells).

**Fig 2:**
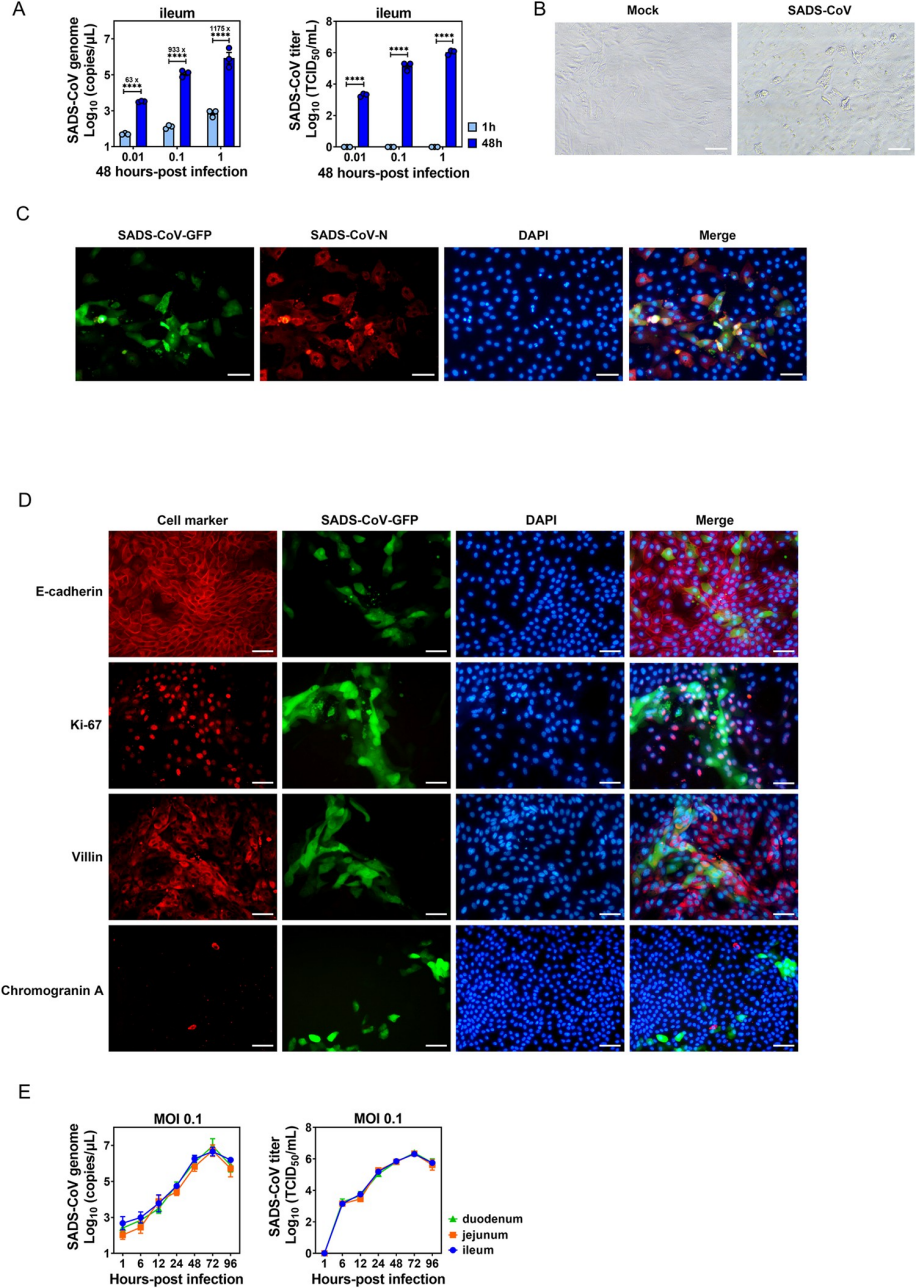
Porcine intestinal enteroid (PIE) cultures support SADS-CoV replication. (A) Ileal PIE monolayers were incubated with medium or SADS-CoV-GFP at the indicated MOIs on a rotary shaker for 1 h at 37°C. The incubated monolayers were washed three times with PBS and harvested at 1 or 48 hpi. Supernatant RNA was extracted and the number of SADS-CoV-GFP genome copies was determined by RT-qPCR and viral titer was determined by a standard TCID_50_ assay. (B) Cytopathic effect (CPE) was observed in ileal PIEs infected with SADS-CoV at 48 hpi (scale bar, 50 μm). (C) Immunofluorescence assay (IFA) was performed using rabbit anti-SADS-CoV-N polyclonal antibody and Alexa Fluor 594-conjugated anti-rabbit IgG as secondary antibody, and nuclei (blue) were visualized by DAPI (scale bar, 50 μm). (D) Colocalization of SADS-CoV-GFP and cellular markers (red) including E-cadherin (epithelial tight junction), Ki-67 (proliferating cells), villin (enterocytes) and chromogranin A (enteroendocrine cells), and nuclei (blue) stained by DAPI (scale bar, 50 μm). (E) Duodenal, jejunal and ileal PIE monolayers were incubated with SADS-CoV-GFP at MOI = 0.1 and the SADS-CoV-GFP replication kinetic was determined by RT-qPCR or TCID_50_. Data are from three independent experiments. *P* values were determined by unpaired two-tailed Student’s t test. *: *p* < .05; **: *p* < .01; ***: *p* < .001; ****: *p* < .0001; ns, not significant.

To evaluate whether 2D PIEs support SADS-CoV replication, we inoculated monolayers of duodenal, jejunal or ileal PIEs with a recombinant SADS-CoV expressing green fluorescent protein (GFP) under different Multiplicity of infections (MOIs) (0.01, 0.1 and 1). In ileal PIE, compared with 1 h post-inoculation (hpi), at 48 hpi the viral genome increased by 63-, 933- and 1175-fold and the virus titer reached 3.3, 5.14 and 6.02 log_10_, respectively, in an MOI-dependent manner (Fig 2A). A similar magnitude of replication was observed in duodenal and jejunal PIEs (S1D Fig). Cytopathic effect (CPE) such as cell rounding and syncytium formation were observed in SADS-CoV-inoculated ileal PIE cultures at 48 hpi (Fig 2B). Viral replication was further demonstrated by co-localization of the viral nucleocapsid N protein with nonstructural secreted GFP in infected cells by immunofluorescent assay (IFA) (Fig 2C). GFP signals were predominantly co-localized with cellular markers of E-cadherin, Ki-67 and villin, but not with chromogranin, indicating that SADS-CoV primarily infects and replicates in enterocytes instead of enteroendocrine cells (Fig 2D). Additionally, SADS-CoV infection exhibited comparable multi-step growth kinetics in all three PIE cultures, which showed a time course-dependent increase both in genomic RNA copies and infectious viral titers until a plateau was reached at 72 hpi (Fig 2E). Taken together, these results indicate that SADS-CoV infection of duodenal, jejunal or ileal PIEs results in an indiscriminately productive viral replication, and this novel infectious model can be used to investigate the relationship between microbiota-derived metabolites and SADS-CoV infection.

### BAs promote SADS-CoV replication in a virus- and cell-specific manner, and affect the early stage of viral infection in PIEs

**Fig 3:**
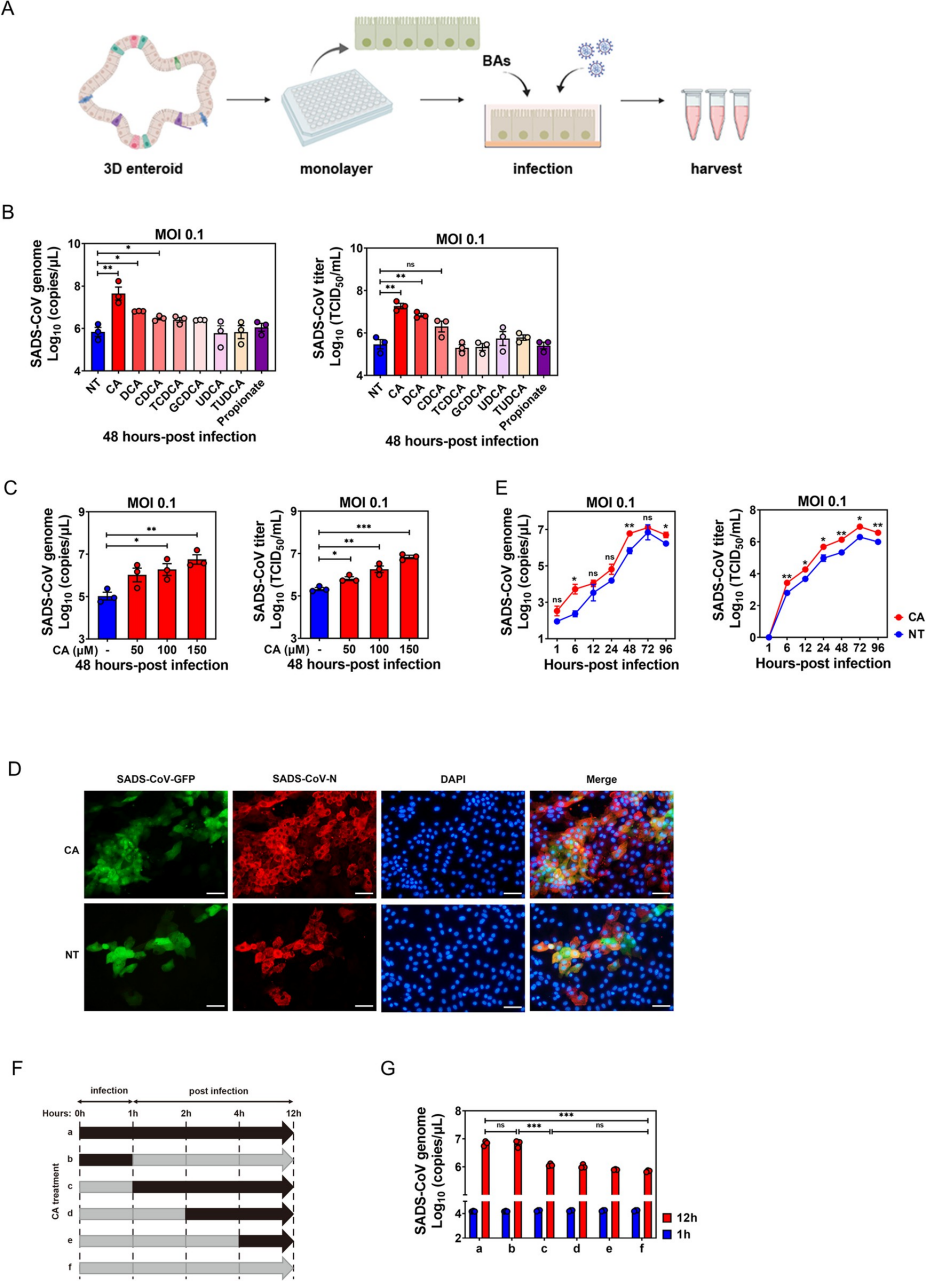
Bile acids (BAs) promote SADS-CoV replication and affect the early stage of viral infection in PIEs. (A) Schematic illustration of BA treatment in SADS-CoV-GFP infection. The image was created using the website https://app.biorender.com/. (B) Ileal PIE monolayers were inoculated with SADS-CoV-GFP at MOI = 0.1 alone or simultaneously with BAs for 48 h at 37°C. All BAs were added to a final concentration of 100 μM, and propionate control to 1 mM. Viral genomic copies and titer were quantified by RT-qPCR and TCID_50_. (C) PIE monolayers infected with SADS-CoV-GFP for 48 h in the presence of different concentrations of cholic acid (CA). (D) Colocalization of SADS-CoV-GFP (green) and SADS-CoV-N protein (red) in CA-treated PIEs and non-treated (NT) controls. (E) The growth kinetics of SADS-CoV-GFP in the presence or absence of 100 μM CA in PIEs. (F) Schematic showing various time periods of CA treatment (black arrows) during infection of PIEs. (G) Virus replication was quantified by RT-qPCR at 1 and 12 hpi. Data are from three independent experiments; *P* values were determined by unpaired two-tailed Student’s t test. *: *p* < .05; **: *p* < .01; ***: *p* < .001; ns, not significant.

Based on our metabolomic profiling observations (Fig 1D–1F), we hypothesized that BA administration would enhance SADS-CoV replication in small intestinal enterocytes, as is the case for PEC, HuNoVs and PEDV [8,11,14]. To test this, we evaluated the efficacy of individual BAs in promoting SADS-CoV replication by adding two unconjugated primary BAs, three conjugated BAs and two unconjugated secondary BA to 2D ileum PIEs simultaneously with SADS-CoV inoculation (Fig 3A). All BAs were tested at 100 μM in the experiment and showed no cytotoxic effect to PIEs at this dose (S2A Fig).

Indeed, CA supplementation resulted in an ~65-fold increase in viral genomic copies or approximately 63-fold augmentation in infectious titers at 48 hpi (Fig 3B). DCA- or CDCA-treated PIEs also exhibited increased viral titers compared to the non-treated control (NT), albeit to a lesser extent than the CA-treated ileal PIE cultures (Fig 3B). In contrast, TCDCA, GCDCA, UDCA or TUDCA barely supported SADS-CoV replication (Fig 3B). Since KEGG analyses also showed that the differentially decreased metabolites in the small intestines of infected piglets were largely associated with carbohydrate metabolism (Fig 1C), we chose propionate, a common short-chain fatty acid that is produced by commensal bacteria-associated carbohydrate fermentation, as an unrelated control that showed no enhancing effect on SADS-CoV replication (Fig 3B).

As previously described, BAs displayed an enhancing effect on PEDV infectivity, but had a negative influence on PDCoV replication in swine intestinal cell lines [7,14]. Despite these seemingly contradictory observations, it was of great interest to test whether BA-stimulated SADS-CoV replication in PIEs generally extends to other SECoVs. Interestingly, CA supplementation hardly elevated PEDV viral genomic RNA level in ileal PIEs at 24 hpi (S2B Fig), suggesting that BAs may act very differently in modulating the replication of distinct SECoVs in PIEs.

To test whether BA-directed promotion of SADS-CoV replication is a unique phenomenon only observed in intestinal epithelial cells, we next repeated these experiments in the porcine intestinal cell line IPEC-J2 and swine testis cell line ST, which are permissive to SADS-CoV infection. All BAs were tested at a noncytotoxic dose of 150 μM in the experiments. Mirroring the results from our initial studies in PIEs, each of the BAs except for GCDCA caused a marked increase in viral genomic RNA copies compared to NT and propionate controls at 24 hpi (S2C Fig, left panel). However, in ST cells, CDCA and TCDCA exerted an opposite effect, dampening viral replication and reducing viral titers by 31.8- and 7.5-fold, respectively, compared to the controls at 24 hpi. GCDCA or DCA treatment had no effect on virus titers (S2C Fig, right panel). Collectively, these data indicate that BAs may regulate distinct features of virus replication in a cell type-specific manner.

Since CA had the strongest enhancing effect on viral replication among all tested BAs, we chose it for further studies on the mechanism(s) of how BAs act on intestinal epithelial cells to facilitate SADS-CoV replication. As expected, CA supported SADS-CoV replication in a dose-dependent manner (Fig 3C). Furthermore, CA enhanced SADS-CoV replication in duodenal and jejunal PIEs (S2D Fig), indicating that CA has no regional effects on enhancing SADS-CoV replication in PIEs. Consistent with previous results, a marked increase in GFP and viral N protein was observed in CA-treated PIEs compared to NT controls (Fig 3D). To further confirm the promoting effect exerted by CA on SADS-CoV replication, we evaluated the growth kinetics of SADS-CoV infection at MOI = 0.1 with or without CA treatment. An obvious increase in viral genomic RNAs and infectious viral titers was seen at each time point from 6 to 72 hpi in CA-treated PIEs compared to the NT control (Fig 3E).

To further dissect the specific phase in which CA acts during the SADS-CoV replication cycle, a time-course study was carried out looking at the effect of BA administration on viral replication at 12 hpi (Fig 3F). Addition of CA at 0 hpi (treatment a) resulted in a marked increase in viral replication 12 h later, compared to 1 hpi or NT control (treatment f). Adding CA at 0 hpi and incubating for only 1 h (treatment b) was sufficient to obtain a comparable enhancement of viral replication, whereas addition CA at 1 (treatment c), 2 (treatment d) or 4 hpi (treatment e) had no effect on viral replication enhancement (Fig 3G). These results indicate that CA functions at a very early stage of viral infection to promote SADS-CoV replication, implying that it may facilitate virus binding or entry into cells.

### BA-associated enhancement of SADS-CoV replication is not dependent on BA receptor signaling, innate immune pathways or viral binding

**Fig 4:**
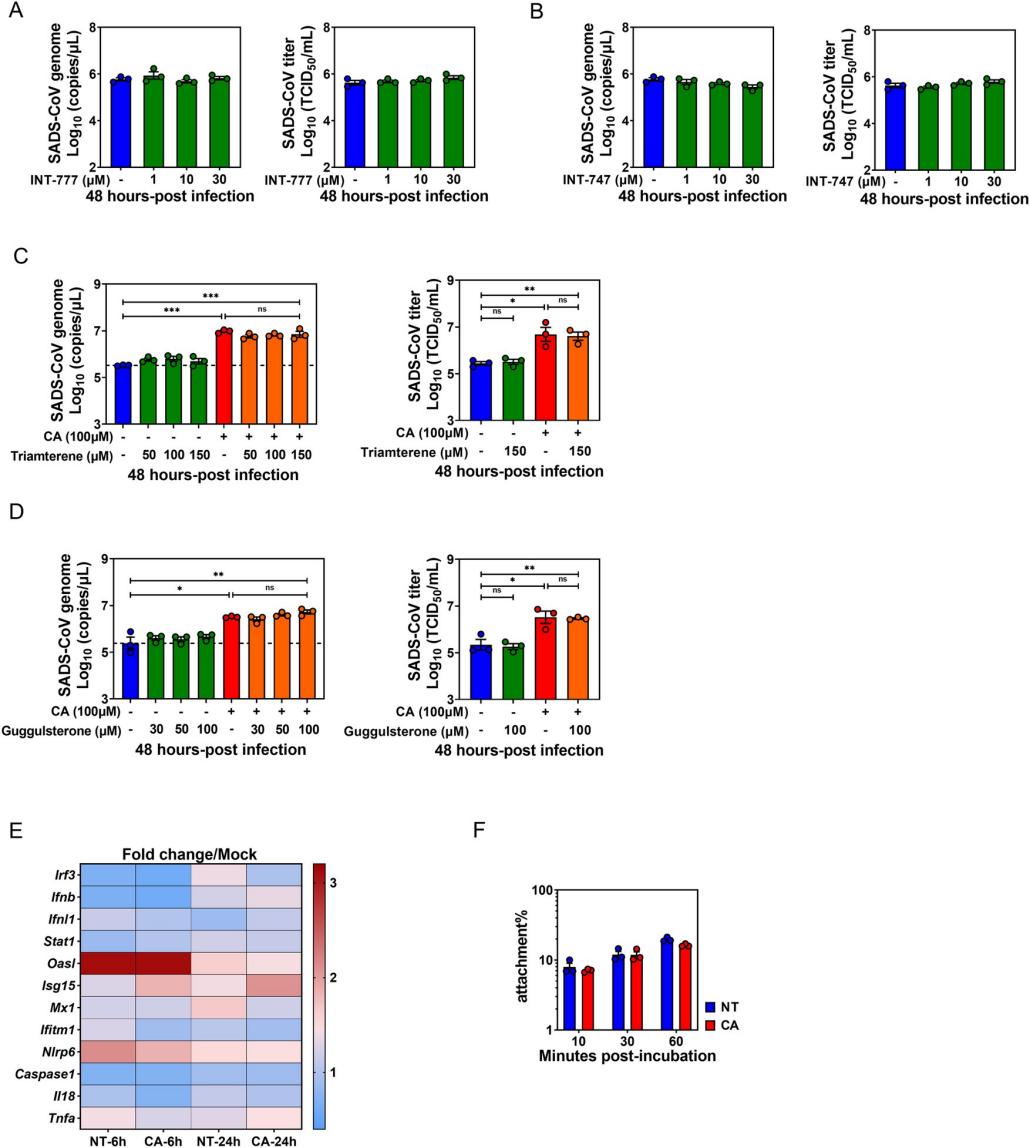
Bile acid (BA)-associated enhancement of SADS-CoV replication is not dependent on BA receptor signaling, innate immune regulation or viral binding. Porcine intestinal enteroid (PIE) monolayers were infected with SADS-CoV-GFP at an MOI = 0.1 for 48 h. (A) TGR5 agonist INT-777 or (B) FXR agonist INT-747 was added to medium during and post-infection at the indicated concentrations. PIE monolayers were pretreated with (C) TGR5 antagonist triamterene or (D) FXR antagonist guggulsterone for 2 h, and then antagonists and cholic acid (CA) were added to the medium at the indicated concentrations during and after SADS-CoV-GFP infection for 48 h. (E) Expression of innate immune-associated antiviral response genes in PIE monolayers infected with SADS-CoV-GFP in the presence or absence of CA at 6 and 24 hpi. Gene expression was determined by qRT-PCR. (F) PIE monolayers were incubated with SADS-CoV-GFP or simultaneously with CA for the indicated time points at 4°C. Viral genome copies were quantified from unwashed cells to determine the amount of input virus and from washed cells to determine the amount of cell-attached virus. Data are reported as the percent of viral genomes remaining cell-associated compared with input. Data are from three independent experiments.

One mechanism by which microbiota-derived metabolites regulate enteric virus infection is to skew the antiviral innate immune response. BAs are known to act on two major receptors: the membrane receptor, Takeda G protein-coupled receptor 5 (TGR5); or nuclear farnesoid X receptor (FXR) [28]. Cumulative evidence has demonstrated that BA signaling via TGR5 or FXR is linked to an anti-inflammatory response involving suppression of NF-κB activity and results in attenuated induction of proinflammatory cytokines in macrophages and monocytes [29,30]. To answer the question of whether CA-treated PIEs have an impaired capacity to limit SADS-CoV replication due to diminished antiviral innate immune responses via TGR5 or FXR signaling pathways, we first treated PIEs with different doses of TGR5 or FXR agonists (INT-777 or INT-747, respectively). No altered viral genomic RNA copies or increase in infectious viral progeny was observed upon SADS-CoV inoculation. Additionally, experiments were carried out treating PIEs with different doses of TGR5 antagonist triamterene or FXR antagonist guggulsterone, then subsequently treated with CA and infected with SADS-CoV. Comparable viral titers were seen between receptor antagonist-treated and untreated PIEs in the absence or presence of CA, implying that TGR5 or FXR signaling is dispensable for SADS-CoV replication or CA-mediated augmentation of viral replication (Figs 4A–4D and S3A and S3B).

Next, we evaluated the expression of genes associated with innate immunity antiviral responses in SADS-CoV-infected PIEs, with or without CA treatment at 6 and 24 hpi. However, no significant reduction in the expression of these genes was observed in the CA-treated PIEs compared to NT controls following SADS-CoV infection (Fig 4E), indicating that CA-driven enhancement of SADS-CoV replication is likely unrelated to innate immunity suppression.

As Nelson *et al*. reported that BAs are cofactors that enhance MNV cell-binding and infectivity [13], we next tested whether CA-mediated stimulation could be attributed to enhanced viral attachment to PIEs. No elevated percentage of viral attachment was observed at any of the time points in CA-treated PIEs compared to NT controls (Fig 4F). In summary, despite acting on PIEs at an early phase of SADS-CoV infection, it is unlikely for BAs to promote viral replication by skewing antiviral innate immune responses through TGR5 or FXR signaling or altering viral attachment.

### BA-mediated enhancement of SADS-CoV replication is dependent on lipid rafts

**Fig 5:**
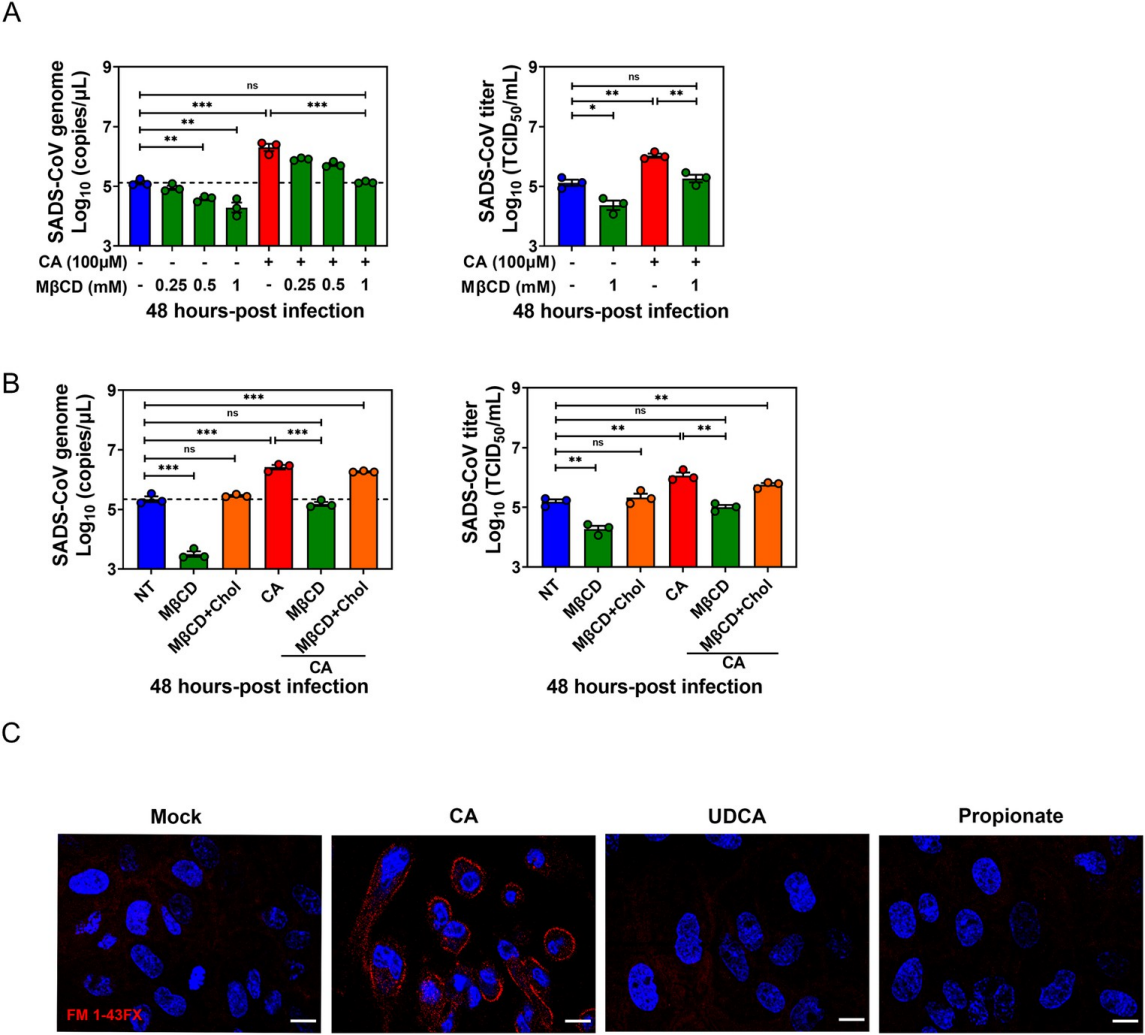
Bile acid (BA) promotion of SADS-CoV replication is dependent on lipid rafts. (A) Porcine intestinal enteroid (PIE) monolayers were pretreated with MβCD for 1 h, and then MβCD and cholic acid (CA) were added to the medium at the indicated concentration during and after SADS-CoV-GFP infection for 48 h. (B) PIE monolayers were pretreated with 1 mM MβCD for 1 h and supplemented with 1 mM cholesterol for 1 h, and then infected with SADS-CoV-GFP, CA and cholesterol were present during and post SADS-CoV-GFP infection. MβCD and CA-treated monolayers without cholesterol replenishment were set up as control. (C) PIE monolayers were treated with either medium alone, 100 μM CA, 100 μM UDCA or 1 mM propionate. Endocytic vesicles (red) were labeled by FM1-43FX and nuclei (blue) were visualized by DAPI (scale bar, 10 μm). Images were acquired by an LSM880 confocal laser-scanning microscope (Zeiss). Data are from three independent experiments; *P* values were determined by unpaired two-tailed Student’s t test. *: *p* < .05; **: *p* < .01; ns, not significant.

Having excluded a role for CA in the regulation of SADS-CoV attachment (Fig 4F), we next determined whether BAs affect viral entry in some other way. Since previous studies demonstrated that the entry of the avian CoV infectious bronchitis virus (IBV) is lipid raft-associated [31], we hypothesized that BA enhances the interactions between SADS-CoV and lipid rafts in order to facilitate viral entry. Indeed, pre-infection supplementation with methyl-β-cyclodextrin (MβCD), which impairs lipid rafts in the plasma membrane that are essential for membrane invagination and endocytosis, led to a significant decrease of SADS-CoV titers at 48 hpi in a dose-dependent manner. Additionally, the CA stimulatory effect was abrogated by MβCD supplementation, also in a dose-dependent manner (Figs 5A and S3C). To test whether this inhibitory effect was attributed to cholesterol depletion from lipid rafts, PIE monolayers were pretreated with MβCD, supplemented with exogenous cholesterol and subsequently infected with SADS-CoV in the presence or absence of CA. Impairment of the CA stimulatory effect by MβCD pretreatment was restored by cholesterol replenishment (Fig 5B), suggesting that BA-associated virus entry depends on intact lipid rafts and membrane cholesterol-mediated endocytosis.

Next, we further confirmed the role of BAs in modulating endocytosis in PIEs using the lipophilic dye FM1-43FX, which incorporates into the cellular membrane and stains endocytic vesicles migrating from the apical brush border. FM1-43FX staining exhibited a remarkable increase of labeled endocytic vesicles in the presence of CA, whereas no phenotype was observed with UDCA or propionate (Fig 5C), consistent with the finding that UDCA did not promote SADS-CoV replication in PIEs (Fig 3B). Together, these data indicate that CA enhances SADS-CoV replication and is associate with lipid raft and membrane cholesterol-mediated endocytosis.

### BA enhances SADS-CoV entry via caveolae-mediated endocytosis

**Fig 6:**
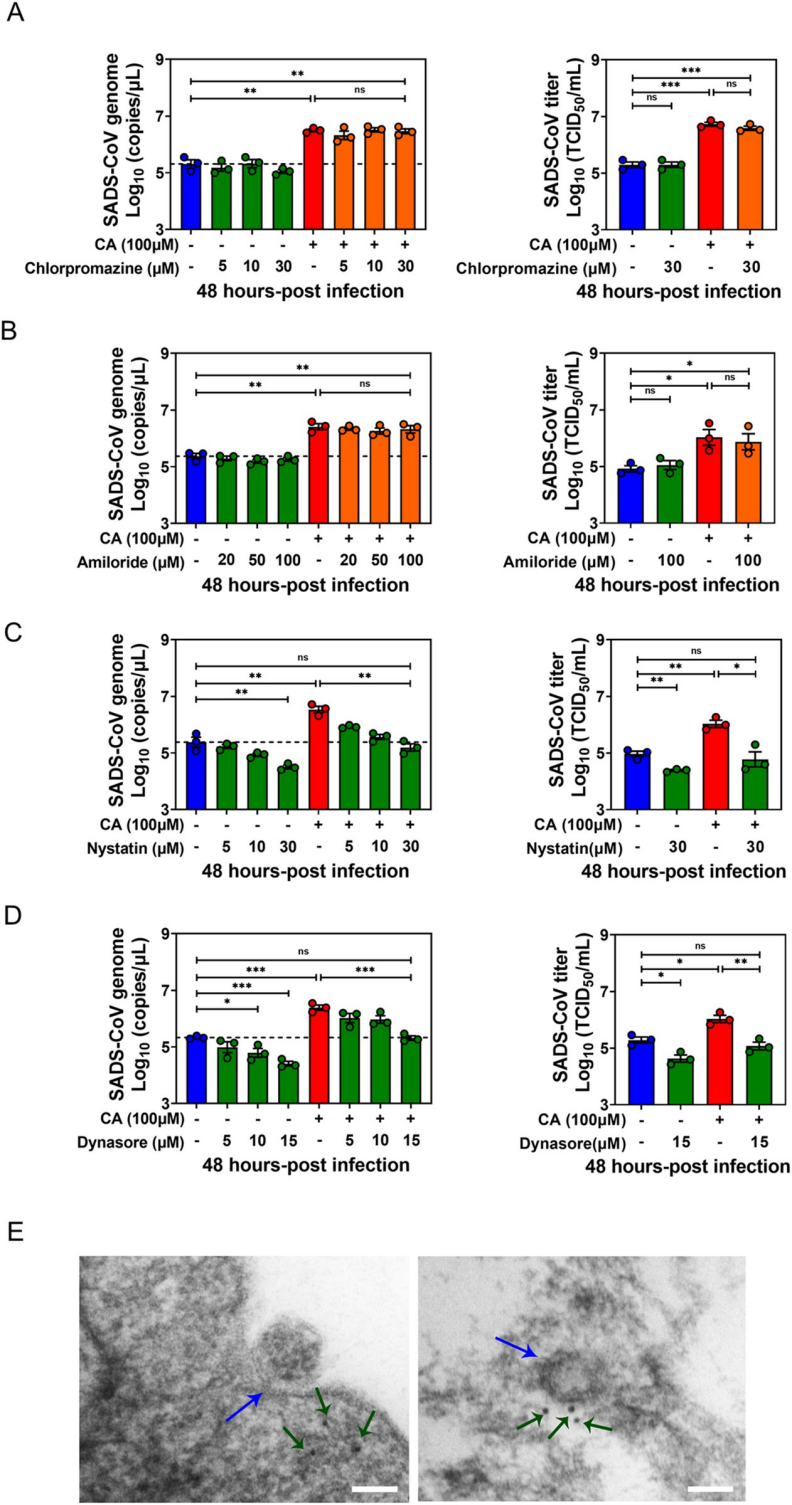
Bile acid (BA) enhances SADS-CoV entry via caveolae-mediated endocytosis. Porcine intestinal enteroid (PIE) monolayers infected with SADS-CoV-GFP in the presence of (A) chlorpromazine (CME inhibitor), (B) amiloride (macropinocytosis inhibitor), (C) nystatin (CavME inhibitor) or (D) dynasore (dynamin 2 inhibitor) at the indicated concentration and harvested at 48 hpi. Chlorpromazine, amiloride, nystatin and dynasore were added during and post-infection; data are from three independent experiments. (E) Caveolin-1 immunogold electron microscopy of SADS-CoV entry. Caveolin-1 was labeled with 10-nm immunogold, as indicated by the green arrow; SADS-CoV is indicated by the blue arrow (scale bar, 100 nm). *P* values were determined by unpaired two-tailed Student’s t test. *: *p* < .05; **: *p* < .01; ***: *p* < .001; ns, not significant.

In mammalian cells, multiple mechanisms are available for the endocytic internalization of virus particles including macropinocytosis, clathrin-mediated endocytosis (CME), caveolae-mediate endocytosis (CavME), and endocytic pathways independent on either clathrin or caveolae [32]. To determine the endocytic pathway on which BAs act to facilitate SADS-CoV replication, we employed diverse pharmacological drugs to block specific endocytic pathways in PIEs and assessed their effects on viral replication. The cytotoxicity of each drug was carefully evaluated by CCK8 assay (S3D Fig). Chlorpromazine (inhibits formation of clathrin-coated pits) or amiloride (specific inhibitor of Na^+^/H^+^ exchanger activity which is fundamental for macropinosome formation) did not hinder SADS-CoV replication or attenuate the stimulatory effect of CA in infected PIEs at 48 hpi (Fig 6A and 6B). The effects of these inhibitors were confirmed with respective controls (transferrin for chlorpromazine and 70kDa dextran for amiloride) (S3E and S3F Fig).

Next, we treated PIEs with a CavME inhibitor, nystatin, and discovered that it blunted both SADS-CoV replication and the CA stimulatory effect in a dose-dependent manner, suggestive of a vital role of CavME in SADS-CoV entry and BA-associated SADS-CoV replication enhancement (Fig 6C). As previously documented, IBV entry is dependent on dynamin 2, which is a GTPase that facilitates membrane fission to generate endocytic vesicles in CME and CavME [31]. To delineate whether dynamin 2 is involved in CA-enhanced endocytosis, we used the specific inhibitor dynasore to block the formation of coated vesicles. Indeed, addition of dynasore diminished both SADS-CoV replication and the CA stimulatory effect in a dose-dependent manner (Fig 6D). Caveolin-1 immunogold electron microscopy (EM) further confirmed the involvement of CavME in SADS-CoV entry in PIEs. Caveolae regions were present on the cell membrane during SADS-CoV invagination (Fig 6E, left panel) and caveolin-1 colocalized with SADS-CoV virions (Fig 6E, right panel). Overall, these results demonstrate BAs may facilitate SADS-CoV entry by influencing CavME, and dynamin 2 is required for this effect.

### BA enhances SADS-CoV replication through endosomal acidification

**Fig 7:**
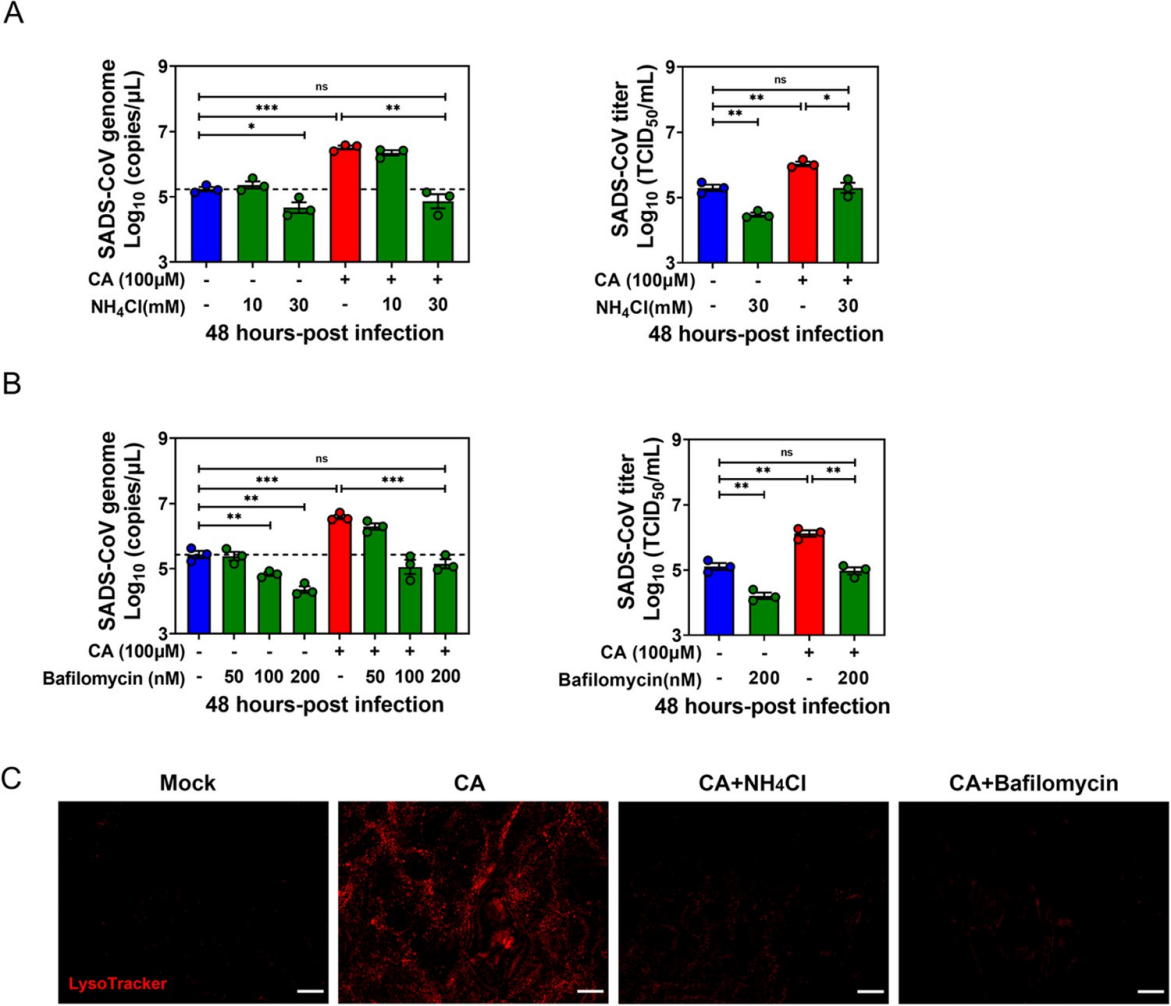
Bile acid (BA) enhances SADS-CoV replication through endosomal acidification. Porcine intestinal enteroid (PIE) monolayers were pretreated with (A) NH_4_Cl and (B) bafilomycin A1 at the indicated concentrations for 1 h before infection, and harvested at 48 hpi. NH_4_Cl and bafilomycin A1 were supplemented during and post-infection. (C) PIE monolayers were treated with either medium alone, 100 μM CA, 100 μM cholic acid (CA) plus 30 mM NH_4_Cl or 100 μM CA plus 200 nM bafilomycin A1 for 1 h, washed with PBS three times and incubated with 200 nM LysoTracker for 30 min at 37°C (scale bar, 10 μm). Images were collected on an LSM880 confocal laser-scanning microscope (Zeiss). Data are from three independent experiments. *P* values were determined by unpaired two-tailed Student’s t test; *: *p* < .05; **: *p* < .01; ***: *p* < .001; ns, not significant.

Viruses depend on the decreasing pH of endocytic organelles as a cue to activate uncoating and penetration into the cytoplasm. Thus, it was necessary to test whether the effect of BA on SADS-CoV entry is dependent on low pH. As shown in Fig 7A, the significant effect of CA treatment on viral genomic RNA and viral titers was abrogated in the presence of the endosome acidification inhibitor NH_4_Cl in a dose-dependent way. We repeated the experiments with bafilomycin A1, a specific inhibitor of vacuolar H^+^-ATPase (V-ATPase) which inhibits endosomal acidification. Consistent with previous studies, viral genomic RNAs and viral titers were greatly reduced in bafilomycin A1-treated PIEs compared to NT controls with or without CA. The effect of bafilomycin A1 was dose dependent (Figs 7B and S3G). Further, we used LysoTracker, a fluorescent dye for labeling and tracking acidic organelles, to determine whether CA treatment significantly augments acidic endo-lysosomal compartments in PIEs. Unsurprisingly, in PIE monolayers only treated with CA, numerous acidic vesicles (LysoTracker-positive red staining) were distributed throughout the cytoplasm. In contrast, CA-treated PIE cultures supplemented with NH_4_Cl or bafilomycin A1 showed very few LysoTracker-positive signals (Fig 7C). These concordant results indicate an important role for endosomal acidification in CA-stimulated viral internalization at the early phase of infection.

### BA treatment alters the trafficking dynamics of SADS-CoV along the endo-lysosomal system

**Fig 8:**
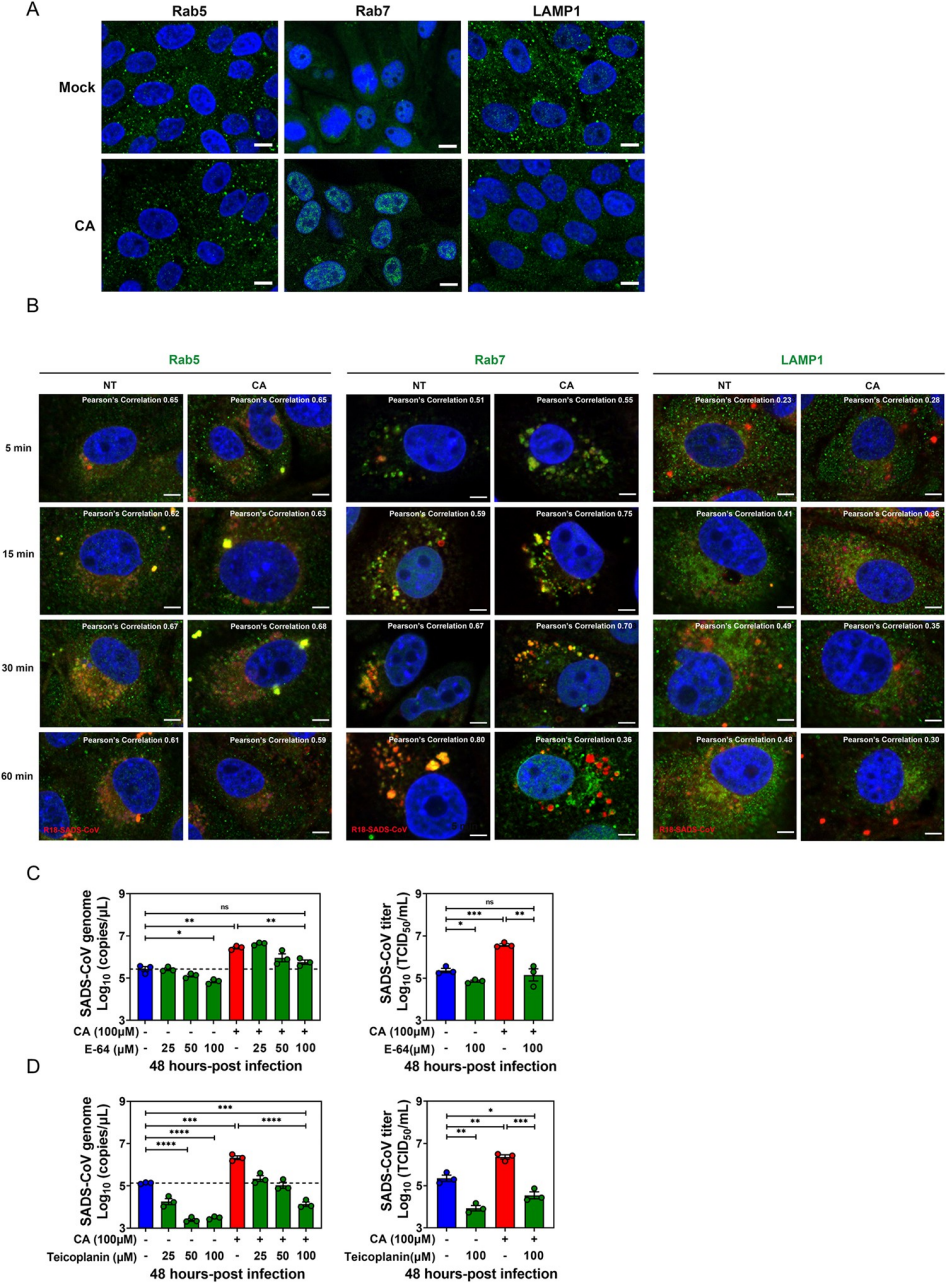
Bile acid (BA) treatment alters the trafficking dynamics of SADS-CoV along the endo-lysosomal system. (A) Porcine intestinal enteroid (PIE) monolayers treated with medium alone or 100 μM cholic acid (CA) for 1 h. Images were acquired by confocal laser-scanning microscopy, detecting Rab5, Rab7 and LAMP1 (green), and nuclei (blue) were visualized by DAPI (scale bar, 10 μm). (B) PIE monolayers were infected with R18-SADS-CoV (red) at an MOI = 20 in the presence or absence of 100 μM CA and incubated at 37°C for 5, 15, 30 or 60 min. The cells were immunostained with Rab5, Rab7, or LAMP1 (green), and nuclei (blue) were visualized by DAPI (scale bar, 5 μm). Images were collected on an LSM880 confocal laser-scanning microscope (Zeiss). Pearson’s correlation coefficient analysis was carried out using Image J software. PIE monolayers were pretreated with cathepsin inhibitor (C) E-64 or (D) teicoplanin for 2 h, and then inhibitors and CA were added to the medium at the indicated concentrations during and after SADS-CoV-GFP infection for 48 h.

We labeled SADS-CoV with the fluorescent lipid R18 as previously described [31] to study the trafficking of SADS-CoV in PIEs during the entry event in the presence or absence of CA. Confocal microscopy was performed with Pearson’s correlation analysis at the indicated time points post virus infection. First, we tested whether CA treatment alone could affect the cellular endo-lysosomal system or not. We demonstrated unaffected expression of Rab5 (marker of early endosomes), greatly enhanced expression of Rab7 (marker of late endosomes) and significantly reduced expression of LAMP1 (marker of lysosomes) in PIEs one hour after CA treatment (Fig 8A). Consistent with these results, the pattern of R18-labeled SADS-CoV (red) and Rab5 (green) colocalization was very similar regardless of CA treatment (Fig 8B, left panel). For Rab7 co-staining, a significant increase in signal appeared in the cytoplasm of infected PIEs as early as 5 min post-infection in the presence of CA, with Rab7 colocalization from 15 to 30 min post-infection. Importantly, colocalization of R18-positive signal with Rab7 was lost by 60 min post-infection only in CA-treated PIEs (Fig 8B, middle panel, Pearson’s correlation 0.80 versus 0.36). Furthermore, while R18-signal in the NT controls colocalized with LAMP1 at 60 min, the majority of virus in the CA treatment was found separate from LAMP1 (Fig 8B, right panel, Pearson’s correlation 0.48 versus 0.30).

Protease cleavage of the S protein is of vital importance for CoV activation and entry, and lysosomal cathepsins are critical for this process during endocytosis. To explore whether cathepsins are required for CA-mediated enhancement of SADS-CoV entry, the PIEs were treated with E-64 (broad inhibitor of cathepsin B, H, L and calpain) or teicoplanin (cathepsin L inhibitor), treated with CA and infected with SADS-CoV. Both E-64 and teicoplanin reduced viral replication at 48 hpi in a dose-dependent manner. A similar phenotype was observed for both cathepsin inhibitors in the presence of CA, suggesting that cathepsins are essential for CA-mediated enhancement of SADS-CoV endocytosis (Figs 8C and 8D and S3H).

Collectively, these data suggest that SADS-CoV moves along the entire endo-lysosomal system in PIEs while BAs alters the dynamics of viral fusion with late endosomal/lysosomal membrane and likely aid subsequent release of SADS-CoV genome (viral uncoating) into the cytosol.

## Discussion

**Fig 9:**
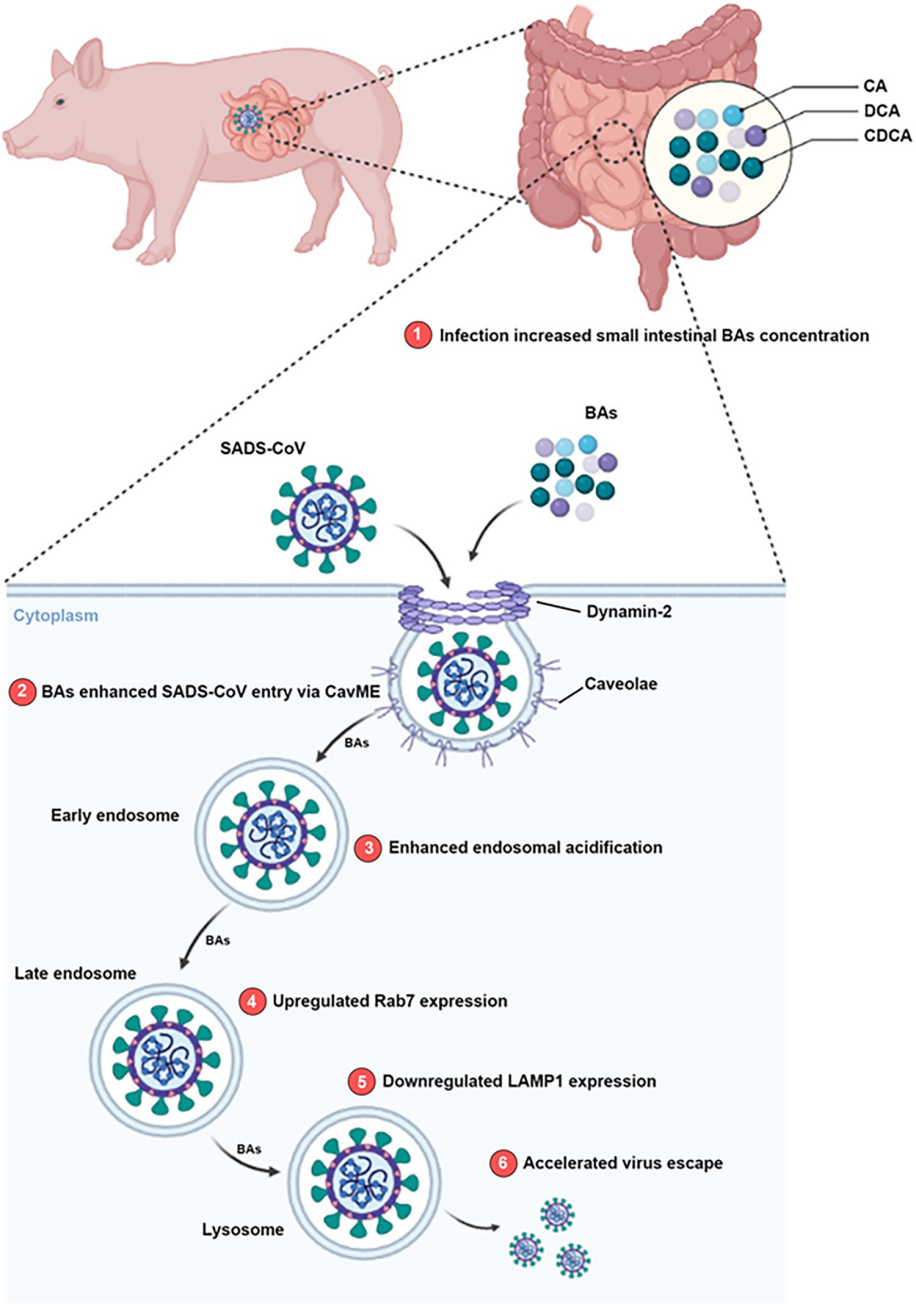
Proposed model for SADS-CoV infection in porcine intestinal enteroids (PIEs) enhanced by bile acids (BA). 1) SADS-CoV infection induces a significant increase of BA concentration in the small intestine of piglets, and this BA-rich microenvironment promotes SADS-CoV replication in the epithelial cells. 2) BAs enhance SADS-CoV entry via CavME, and dynamin-2 is required for this effect. In the presence of BAs, 3) endosomal acidification is increased; 4) Rab7 expression is upregulated and 5) LAMP1 expression is downregulated. These cellular changes induced by BAs might alter the dynamics of endosomal/lysosomal system and 6) aid SADS-CoV escape into the cytoplasm. The image was created using the website https://app.biorender.com/.

Cumulative evidence suggests a fundamental role of the microbial metabolites in regulating viral infections locally and systemically [3,4,33]. Among the immensely diverse microbiota-derived metabolites, BAs not only facilitate nutrient absorption, but also act as pleotropic signaling molecules that modulate mucosal homeostasis and inflammatory responses [23,28]. In the present study, we demonstrated that infection by the potentially zoonotic SADS-CoV in suckling piglets causes significant alteration in the metabolomic profile of intestinal BAs. We established an infection model in PIEs, which recapitulate the structural and functional features of natural intestinal epithelium *in vitro*. With this powerful *ex vivo* tool, we demonstrated that SADS-CoV utilizes several BAs to facilitate entry and infection. Using CA as a model, we propose that BAs act instantly at early stage of SADS-CoV infection to promote CavME and endosomal acidification while altering the dynamics of the endosomal/lysosomal system, which ultimately benefits SADS-CoV replication (Fig 9).

Generally, SADS-CoV infection induced augmentation of BAs in the small intestine of infected piglets compared to control animals (Fig 1D–1F). However, this phenotype was not evenly observed along the small intestines. For instance, in the context of SADS-CoV infection, primary unconjugated CA (which exerted the strongest enhancing effect on SADS-CoV replication; Fig 3B) was more concentrated in the distal (ileum) than the proximal small intestine (duodenum). In contrast, the concentration of secondary conjugated TUDCA (which had no effect on viral replication; Fig 3B) was higher in the proximal than the distal small intestine (Fig 1E). This phenotype agrees with the study conducted by Sun et al., in which the viral load in the ileum was reported to be significantly higher than that of duodenum, suggestive of an intestinal regionalization of SADS-CoV infection [25]. Because SADS-CoV replicated to a comparable level in PIEs created from duodenum, jejunum and ileum (Fig 2E), it is very unlikely that the epithelial cells from different small intestinal regions would support viral replication differently. Thus, it may be feasible that the intestinal regional preference of SADS-CoV is based on the metabolic profile of BAs that render particular stretches of the gut microenvironment beneficial for its replication.

We evaluated the ability of individual BAs to support SADS-CoV replication and found that CA was the most effective, while DCA and CDCA stimulated SADS-CoV replication to a lesser extent (Fig 3B). LCA, the corresponding unconjugated secondary BA converted from CDCA by commensal bacteria, was not included due to inevitable cytotoxicity in PIEs even at a dose of 10 μM (S3I Fig). On the contrary, conjugated primary BAs such as TCDCA and GCDCA, as well as conjugated and unconjugated secondary BAs like TUDCA and UDCA had no effect on viral replication (Fig 3B). These findings are not fully consistent with the work published by Murakami et al., in which most conjugated and unconjugated primary BAs are effective in supporting HuNoV GII.3 replication in HIEs whereas secondary unconjugated BAs were less or not effective [11]. These discrepancies might be explained by two reasons: 1) SADS-CoV and HuNoV GII.3 may benefit from different BAs due to inherent differences in their respective intestinal enteroid cultures, porcine versus human; or 2) GCDCA was tested at 500 μM in HIEs, but could only be tested at 150 μM in PIEs because of cytotoxic effects. Therefore, low tolerable subtoxic dose might be a limitation for GCDCA to show stimulation on SADS-CoV replication in PIEs.

The notion that BAs are active signaling metabolites was established by the discovery of FXR and TGR5 as dedicated BA receptors. In our model, we found that CA-driven enhancement of SADS-CoV replication in PIEs was not altered by treatment with several agonists (INT-747 for FXR, INT-777 for TGR5) or antagonists (guggulsterone for FXR, triamterene for TGR5). Thus, the stimulatory effect exerted by CA does not seem to depend on signaling by the classic BA receptors (Fig 4A–4D). This result is similar to the lack of requirement for FXR or TGR5 signaling in the BA-associated enhancement of PoSaV replication in LLC-PK1 cells [8]. Our finding is also consistent with a study showing BA-dependent HuNoV GII.3 replication in HIEs is not mediated by FXR or TGR5 receptor signaling, but involves a third receptor sphinogosine-1-phosphate receptor 2 (S1PR2) [11]. Additional studies are needed to evaluate the roles of other BA receptors including S1PR2, Pregnane X receptor, constitutive androstane receptor and vitamin D receptor [28] in the CA-mediated enhancement of SADS-CoV replication in PIEs.

As active mediators of innate immunity, BAs are recognized to have indirect proviral activity by skewing the innate antiviral responses in host cells. For example, Chang and George demonstrated that adding individual BAs onto hepatitis C virus replicon-harboring Huh-7 cells promoted viral replication and reduced the antiviral effect of IFN in an FXR-dependent manner [34]. In addition, BAs were shown to enhance PEC replication in LLC-PK1 cells by inhibiting cellular innate immunity via reduction of STAT1 phosphorylation [9]. Our model is inconsistent with these two studies, as we observed barely significant downregulation of IFN-related gene expression in the CA-treated PIEs vs NT controls upon SADS-CoV infection (Fig 4E), suggesting that the innate immune responses in enterocytes might be intrinsically less sensitive to BA exposure.

With our time-of-addition study, we were able to show that the first hour of infection was critical for CA to exert its stimulating effect on viral replication (Fig 3G). We also found that CA-stimulation of viral replication was lipid raft associated, as determined by MβCD disruption and cholesterol replenishment experiments. This finding was consistent with the research demonstrating lipid rafts are necessary for IBV entry into host cells [31]. Lipid rafts are not only organizing centers for assembly of signaling molecules, but also a framework where viral structural proteins bind to cellular receptors [32]. Human immunodeficiency virus utilizes highly concentrated glycosphingolipid galactosyl-ceramide (GalCer) at lipid rafts on the surface of epithelial cells as an alternative receptor for cell entry [35]. However, it does not seem that BAs enhance SADS-CoV penetration by creating a receptor-rich region on the lipid rafts to facilitate its initial binding (Fig 4F).

Next, the effect of nystatin demonstrated that CA promotes SADS-CoV internalization by enhancing CavME (Fig 6C). For the first time, we identified that SADS-CoV penetrates intestinal epithelial cells through CavME. Our data are consistent with previous studies demonstrating that human CoV 229E enters host cells through CavME, and PEDV is known to employ both clathrin-mediated endocytosis (CME) and CavME for entry [36,37]. Moreover, a crucial role for dynamin 2 was found in CA-stimulated SADS-CoV entry (Fig 6D). Further experiments showed that use of chemical inhibitors including NH_4_Cl and bafilomycin A1 to oppose endosome acidification in PIEs resulted in the neutralization of CA-mediated enhancement of SADS-CoV replication (Fig 7A and 7B), suggesting that the low pH in endosomes is required for CA to promote SADS-CoV replication. These results are similar to former studies showing a dependence on low pH for endocytosis of PEDV, and a highly neurovirulent CoV, porcine hemagglutinating encephalomyelitis virus (PHEV) [38,39]. One limitation of these studies is that the importance of these mediators in viral entry was based only on experiments with chemical inhibitors. Despite multiple attempts, we were unable to use siRNA to efficiently knockdown the respective genes in PIEs, nor was it possible to generate knockout PIE cell lines.

In summary, our mechanistic study using a novel *ex vivo* PIE infectious model demonstrates how cellular biological events are modulated by BAs to facilitate SADS-CoV entry and subsequent replication in PIEs. The finding that BAs can enhance SADS-CoV endocytosis via CavME and endosomal acidification, while altering the dynamics of the endosomal/lysosomal system. This study provides insights into how SECoVs exploit the intestinal microenvironment to rapidly establish infection and invade the epithelial barrier, and will open new approaches for development of antiviral therapies against SADS-CoV infection.

## Materials and methods

### Ethics statement

All experiments described in this study were reviewed and approved by the Experimental Animal Ethics Committee of Zhejiang University (approval no. ZJU20170026).

### Cells and viruses

Vero cells, IPEC-J2 and ST cells were cultured in Dulbecco’s modified Eagle’s medium (DMEM, HyClone) supplemented with 10% (v/v) fetal bovine serum (FBS, Gibco) in the presence of 100 U/mL penicillin and 100 U/mL streptomycin under 37°C, 5% CO_2_, and water-saturated humidity conditions.

The SADS-CoV isolate CH/GD-01/2017 p10 was used in the animal experiment and R18 labeled virus experiment [19,40]. SADS-CoV expressing green fluorescent protein (GFP) was used in the PIEs infection. The PEDV virulent strain ZJU/G2/2013 (GenBank accession no. KU558701) was also used in the study [41,42]. SADS-CoV and PEDV viral titers were determined in Vero cells by endpoint dilution as the 50% tissue culture infective dose (TCID_50_).

### Bile acids, agonists and chemical inhibitors

All BAs were purchased from MedChemExpress (Monmouth Junction, NJ, USA) and dissolved in dimethyl sulfoxide (DMSO, Sigma Aldrich). The FXR agonist INT-747 (HY-12222) and antagonist guggulsterone (HY-107738), TGR5 agonist INT-777 (HY-15677) and antagonist triamterene (HY-B0575) were purchased from MedChemExpress. Cellular behaviors in the presence of BAs were studied by using MβCD (HY-101461) and bafilomycin A1 (HY-10058) purchased from MedChemExpress. Chlorpromazine (S2456), amiloride (S1811), nystatin (S1934) and dynasore (S8047) were purchased from Selleck. Cholesterol-water soluble (C4951) and NH_4_Cl (A9434) were purchased from Sigma Aldrich. Cathepsin inhibitor E-64 (E3132) was purchased from Sigma Aldrich and teicoplanin (ab141430) was purchased from Abcam.

### Animal experiments

The live animal experiment was approved by the Experimental Animal Ethics Committee of Zhejiang University (approval no. ZJU20170026). Briefly, twelve 3-day-old conventional piglets, free of SADS-CoV, PEDV, TGEV and PDCoV RNA in the feces, were randomly assigned into two groups. Piglets in each group were housed with their mothers (SADS-CoV RNA and serum antibody negative as determined by IFA) without any artificial supplemental colostrum or milk. One group was challenged orally with a SADS-CoV/CH/GD-01/2017/P10 isolate at a dose of 1 × 10^5^ plaque-forming units (PFU)/mL (3 mL per pig), whereas the other group was orally administrated 3 mL of DMEM as negative controls. All piglets were monitored daily for any signs of illness. Three piglets in each group were euthanized at 7 days post-infection (dpi) while the remaining three in each group were necropsied at 21 dpi. Samples of duodenum, jejunum and ileum were subjected to quasi-targeted metabolomics and LC-MS/MS to determine the concentrations of different bile acids. Fecal swabs for viral RNA detection were collected at 1, 3, 7, 14 and 21 dpi from all pigs until they were sacrificed.

### Quasi-targeted metabolomics

Metabolites were extracted from the porcine intestinal tissue. Briefly, samples were flash-frozen in liquid nitrogen and ground into powder using a sterile mortar and pestle, then resuspended in pre-chilled 80% methanol and vortexed well. Samples were incubated on ice for 5 min and centrifuged at 15000 x g, 4°C for 15 min. The supernatant was injected into the LC-MS/MS system analysis. UHPLC-MS/MS analyses were performed using a Vanquish UHPLC system (Thermo Fisher, Germany) coupled with an Orbitrap Q ExactiveTMHF-X mass spectrometer (Thermo Fisher, Germany) in Novogene Co., Ltd. (Beijing, China). The raw data files generated by UHPLC-MS/MS were processed using the Compound Discoverer 3.1 (CD3.1, Thermo Fisher) to perform peak alignment, peak picking, and quantitation for each metabolite. These metabolites were annotated using the KEGG database (https://www.genome.jp/kegg/pathway.html) and HMDB database (https://hmdb.ca/metabolites). PCA and partial least squares discriminant analysis (OPLS-DA) were performed with metaX (a flexible and comprehensive software for processing metabolomics data).

### LC-MS/MS determination of tissue bile acid concentrations

Small intestine tissue samples were ground in liquid nitrogen, resuspended in 500 μL of acetonitrile/methanol (8:2) and centrifuged at 12,000 x g for 20 min. The supernatant was then dried using a nitrogen blower. The precipitates were reconstituted in 100 μL water/acetonitrile (8:2) with formic acid (0.1%) by thorough vortexing and centrifugation. The final supernatant (2 μL) was injected into the LC-MS/MS system for analysis. An ultra-high performance liquid chromatography coupled to tandem mass spectrometry (UHPLC-MS/MS) system (ExionLC AD UHPLC-QTRAP 6500+, AB SCIEX Corp., Boston, MA, USA) was used to quantitate bile acids at Novogene Co., Ltd. (Beijing, China). LC-MS was used to detect the concentration series of standard solution. The concentration of standard was used as abscissa, and the ratio of internal standard peak area was used as ordinate to investigate the linearity of the standard solution.

### Porcine intestinal crypt isolation and 3D enteroid culture

Porcine intestinal crypts were isolated from 2- to 14-day-old piglets as previously described [26,27]. The intestinal tissues were cut vertically and dissected into 2 mm segments and washed with PBS several times until supernatants were clear. Intestinal segments were resuspended in Gentle Cell Dissociation Reagent (Stem Cell, Canada) and incubated on a rocking platform at 20 rpm for 20 min. Tissue supernatants were collected and resuspended in 0.1% BSA and passed through a 70-μm nylon filter. The crypts were centrifuged at 300 x g for 5 min and resuspended in cold DMEM/F12 (HyClone). After counting, crypts were resuspended with IntestiCult Organoid Growth Medium (STEM CELL) and Matrigel (Corning) and seeded in a 24-well plate. Cell cultures were incubated at 37°C for 10 min until the Matrigel was solidified, and 500 μL IntestiCult Organoid Growth Medium was added into each well.

### 2D porcine intestinal enteroid monolayer culture

Mature enteroids with DMEM/F12 were harvested and centrifuged at 200 x g for 5 min. The supernatant was removed and enteroids were resuspended in 0.05% Trypsin-EDTA (Gibco), incubated at 37°C for 5 min and dissociated by repeated pipetting to obtain a single-cell suspension. DMEM/F12 with 20% (v/v) FBS was added to the suspension and centrifuged at 800 x g for 5 min. The cells were then resuspended with IntestiCult Organoid Growth Medium and seeded into a Matrigel-coated 96-well plate. After 3 days of differentiation, the enteroid monolayers were ready for experimental use.

### Cell cytotoxicity assay

PIE monolayers were incubated in medium alone or with different additives for 1 h at 37°C, then 10 μL CCK-8 reagent (C0037, Beyotime Institute of Biotechnology) was added per well and incubated for 1 h at 37°C. The absorbance values of 96-well plate wells were read at 450 nm; three parallel wells were set for each group, and the mean value was obtained. A cell viability curve was plotted with time as ordinate and optical density (OD) value as abscissa. Cell survival rate was calculated using the following formula: cell survival rate (%) = (OD _the experimental group_/OD _the control group_) × 100%.

### RNA isolation and reverse transcriptase quantitative PCR

Total RNA from cell cultures was extracted using TRIzol reagent (Invitrogen) following the manufacturer’s instructions. Viral RNA titer was determined by a HiScript II one Step qRT-PCR Probe Kit (Vazyme). Genes tested included interferon (IFN) type I (*Ifnb*) and type III (*Ifnl1*), IFN signaling molecules (*Irf3* and *Stat1*), IFN-stimulated genes (ISGs; *Isg15*, *Oas1*, *Mx1* and *Ifitm1*) and NOD-like receptor family pyrin domain-containing protein 6 (NLRP6)-inflammasome (*Nlrp6*, *Caspase1*, *Il1b* and *Il18*). Gene expression was determined by qRT-PCR using the HiScript II one Step qRT-PCR SYBR Green Kit (Vazyme). Primer sequences and probes are listed in S1 Table.

### Immunofluorescence assay

PIE monolayers were grown in glass-bottom 15-mm culture slides or 96-well plates. PIE monolayers were fixed with 4% paraformaldehyde (PFA) for 30 min, permeabilized with 0.5% Triton X-100 for 15 min and blocked with 5% BSA for 1 h. Subsequently, PIE monolayers were probed with primary antibodies overnight at 4°C, stained with the secondary antibodies for 1 h at room temperature and incubated with DAPI for 5 min. The cell markers of intestinal epithelial cells were detected using anti-E-cadherin (Proteintech, 20874-1-AP), anti-Ki-67 (Affinity, AF0198), anti-villin (Proteintech, 16488-1-AP) or anti-chromogranin A (Abcam, ab15160). For endosome and lysosome detection, anti-Rab5 (Proteintech, 11947-1-AP), anti-Rab7 (Proteintech, 55469-1-AP) and anti-LAMP1 (Proteintech, 55273-1-AP) were utilized. The SADS-CoV nucleocapsid N protein was detected by anti-SADS-CoV polyclonal Antibody. For the measurement of endocytosis, FM1-43FX (Thermo, F35355) and LysoTracker (Thermo, L12492) were used as previously described [26,27]. PIE monolayers were treated with medium containing 5 μg/mL of FM1-43FX alone or with indicated additives for 1 h at 37°C, fixed with 4% PFA and stained with DAPI for 5 min. For LysoTracker, PIE monolayers were treated with medium containing the indicated additives for 1 h and incubated with 200 nM LysoTracker for 30 min at 37°C. Images were collected with a LSM880 confocal laser-scanning microscope (Zeiss). For the confirmation of the effectiveness of macropinocytosis, clathrin-mediated endocytosis inhibitors, Alexa-594-labeled transferrin (Invitrogen T13343) and FITC-labeled 70kDa dextran (Sigma Aldrich 46945) were used as previously described [43,44]. For transferrin, PIE monolayers were treated with medium alone or containing 30 μM chlorpromazine for 1 h and incubated with 25 μg/mL Alexa-594-labeled transferrin for 30 min at 37°C, fixed with 4% PFA and stained with DAPI for 5 min. For 70kDa dextran, PIE monolayers were treated with medium alone or containing 100 μM amiloride for 1 h and incubated with 1 mg/mL FITC-labeled 70kDa dextran for 30 min at 37°C, fixed with 4% PFA and stained with DAPI for 5 min. Images were collected with a LSM880 confocal laser-scanning microscope (Zeiss).

### Immunoelectron microscopy

PIE monolayers were first incubated with SADS-CoV at an MOI = 200 for 30 min at 4°C, then transferred to 37°C, fixed with immune electron microscopy fixative (0.1% glutaraldehyde and 3%PFA in 0.1 M PBS) for 2 h at room temperature, and finally collected. The collected cell precipitate was resuspended and washed twice with precooled 0.1 M PBS (pH 7.4). After the supernatant was removed, samples were processed with dehydration, resin penetration, embedding and polymerization steps. Samples were sliced into 90-nm ultrathin cryosections using a Leica UC7 ultramicrotome and collected onto 150-mesh nickel grids for immunogold labeling. The nickel grids were incubated with a 1:20 dilution of rabbit anti-caveolin-1 antibody (A1555, ABclonal) overnight at 4°C. The nickel grids were rinsed with PBS 6 x 3 min and then incubated with a 1:100 dilution of gold-conjugated goat anti-rabbit IgG (G7402, Sigma Aldrich) for 2 h at 28°C. The grids were washed and stained with 2% uranyl acetate. Finally, the sections were examined on a transmission electron microscope (H-7650; Hitachi).

### R18 labeling of SADS-CoV

R18 labeling was performed as previously described [26,27]. Briefly, 100 μL SADS-CoV was incubated with 200 μM R18 (Thermo O246) on a rotary shaker for 1 h at 37°C and resuspended in 10 mL cold PBS. Excess dye was removed with an Amicon Ultra-15 Centrifugal Filter (10 kDa, Millipore) by centrifugation at 4000 x g for 1 h at 4°C.

### Statistical analysis

Statistical analyses were performed with Prism GraphPad software v 9.0. Error bars represent standard errors of the means (SEM) in all figures and *p* values were determined by unpaired, two-tailed Student’s t test. Each experiment was performed three times. *: *p* < .05; **: *p* < .01; ***: *p* < .001; ****: *p* < .0001.

## Supporting information

S1 Fig Porcine intestinal enteroid (PIE) cultures can support SADS-CoV replication. (A) 3D PIEs were derived from small intestinal crypts and cultured in Matrigel. (B) Single cell suspensions from the 3D PIEs were seeded in Matrigel-coated 96-well plates to form 2D enteroid monolayers. (C) PIE monolayers were immunostained for cellular markers (red) including E-cadherin, Ki-67, villin and chromogranin A, and nuclei (blue) were visualized by DAPI (scale bar, 50 μm). (D) Duodenal and jejunal PIE monolayers were inoculated with medium or SADS-CoV-GFP at different MOIs and titrated at 1 or 48 hpi.
(TIF)

S2 Fig Bile acids (BAs) enhance SADS-CoV replication in a virus-specific and cell-specific manner. (A) Cytotoxic effect of different BAs in PIEs at the indicated concentration. (B) PIE monolayers were infected with PEDV at MOI = 1 in the presence of BAs for 24 h. Viral titer was determined by qRT-PCR. (C) IPEC-J2 and ST cells were infected with SADS-CoV-GFP at MOI = 0.1 in the presence of BAs, and viral replication was determined by qRT-PCR at 24 hpi. (D) Duodenal and jejunal PIE monolayers were infected with SADS-CoV at MOI = 0.1 in the presence or absence of cholic acid (CA) for 48 h, and viral replication was determined by qRT-PCR and TCID_50_ assay. Data are from three independent experiments. *P* values were determined by unpaired two-tailed Student’s t test. *: *p* < .05; **: *p* < .01; ***: *p* < .001; ns, not significant.
(TIF)

S3 Fig Cellular viability under the treatment of different chemical additives. Cytotoxic effect of (A) TGR5 agonist INT-777 and FXR agonist INT-747, (B) TGR5 antagonist triamterene and FXR antagonist guggulsterone, (C) MβCD, (D) chlorpromazine (CME inhibitor), amiloride (macropinocytosis inhibitor), nystatin (CavME inhibitor) and dynasore (dynamin 2 inhibitor), in porcine intestinal enteroids (PIEs) at the indicated concentrations. (E) PIE monolayers were treated with either medium alone or 30 μM chlorpromazine for 1 h, washed with PBS three times and incubated with 25 μg/mL Alexa-594-labeled transferrin for 30 min at 37°C (scale bar, 10 μm). Images were collected on an LSM880 confocal laser-scanning microscope (Zeiss). (F) PIE monolayers were treated with either medium alone or 100 μM amiloride for 1 h, washed with PBS three times and incubated with 1mg/mL FITC-labeled 70kDa dextran for 30 min at 37°C (scale bar, 10 μm). Images were collected on an LSM880 confocal laser-scanning microscope (Zeiss). Cytotoxic effect of (G) NH_4_Cl and bafilomycin A1, (H) cathepsin inhibitors E-64 and teicoplanin (I) LCA in porcine intestinal enteroids (PIEs) at the indicated concentrations.
(TIF)

S1 Table Primer sets used for quantitative reverse transcription-PCR. (DOCX)
