# Supplementary figures and images for: Bile acids promote the caveolae-associated entry of swine acute diarrhea syndrome coronavirus in porcine intestinal enteroids
Source: PLoS Pathog. 2022 Jun 13;18(6):e1010620. doi: 10.1371/journal.ppat.1010620 (PMC9249351; doi:10.1371/journal.ppat.1010620)

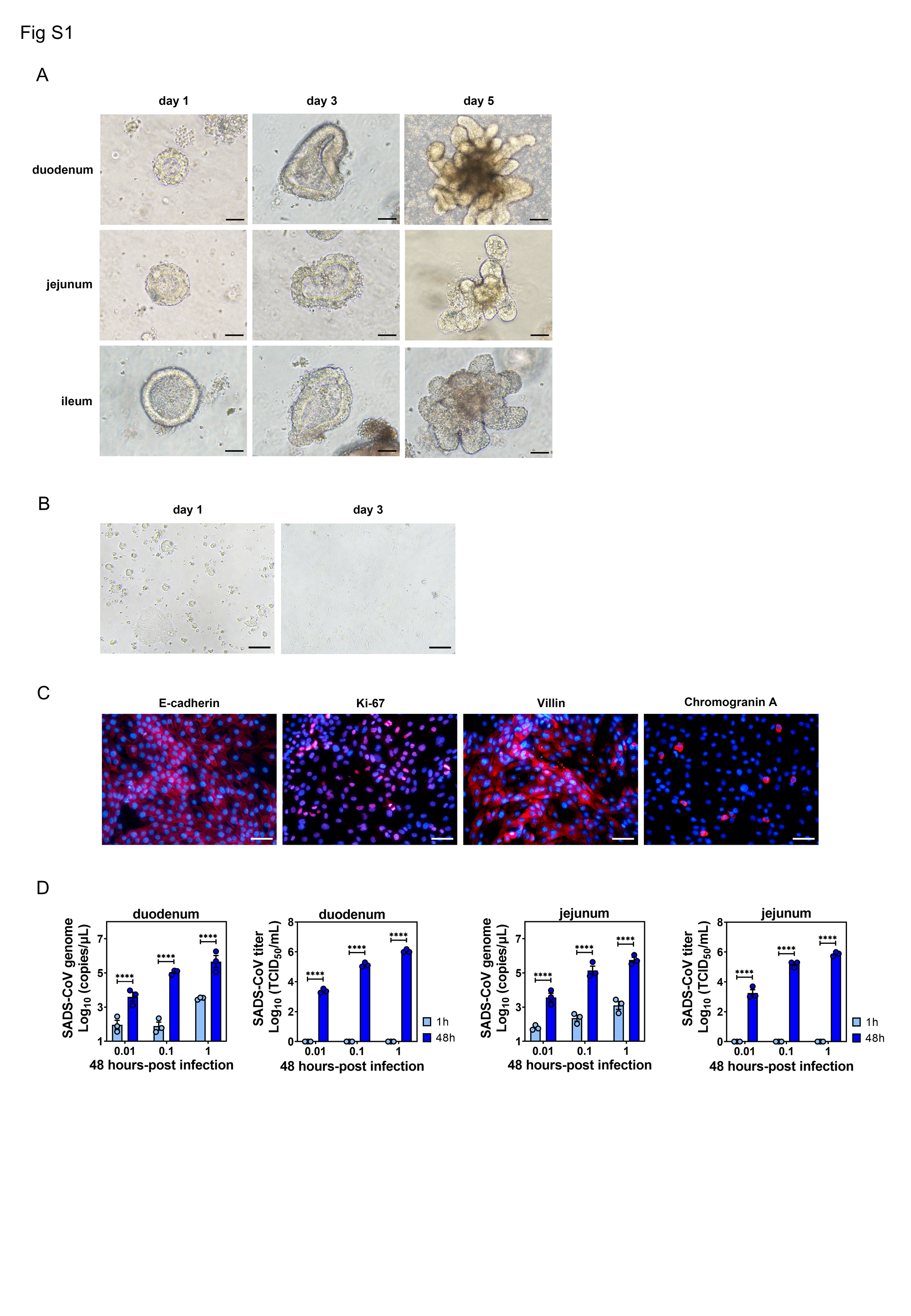

Supplement: S1 Fig — (A) 3D PIEs were derived from small intestinal crypts and cultured in Matrigel. (B) Single cell suspensions from the 3D PIEs were seeded in Matrigel-coated 96-well plates to form 2D enteroid monolayers. (C) PIE monolayers were immunostained for cellular markers (red) including E-cadherin, Ki-67, villin and chromogranin A, and nuclei (blue) were visualized by DAPI (scale bar, 50 μm). (D) Duodenal and jejunal PIE monolayers were inoculated with medium or SADS-CoV-GFP at different MOIs and titrated at 1 or 48 hpi. (TIF) [file ppat.1010620.s001.TIF]

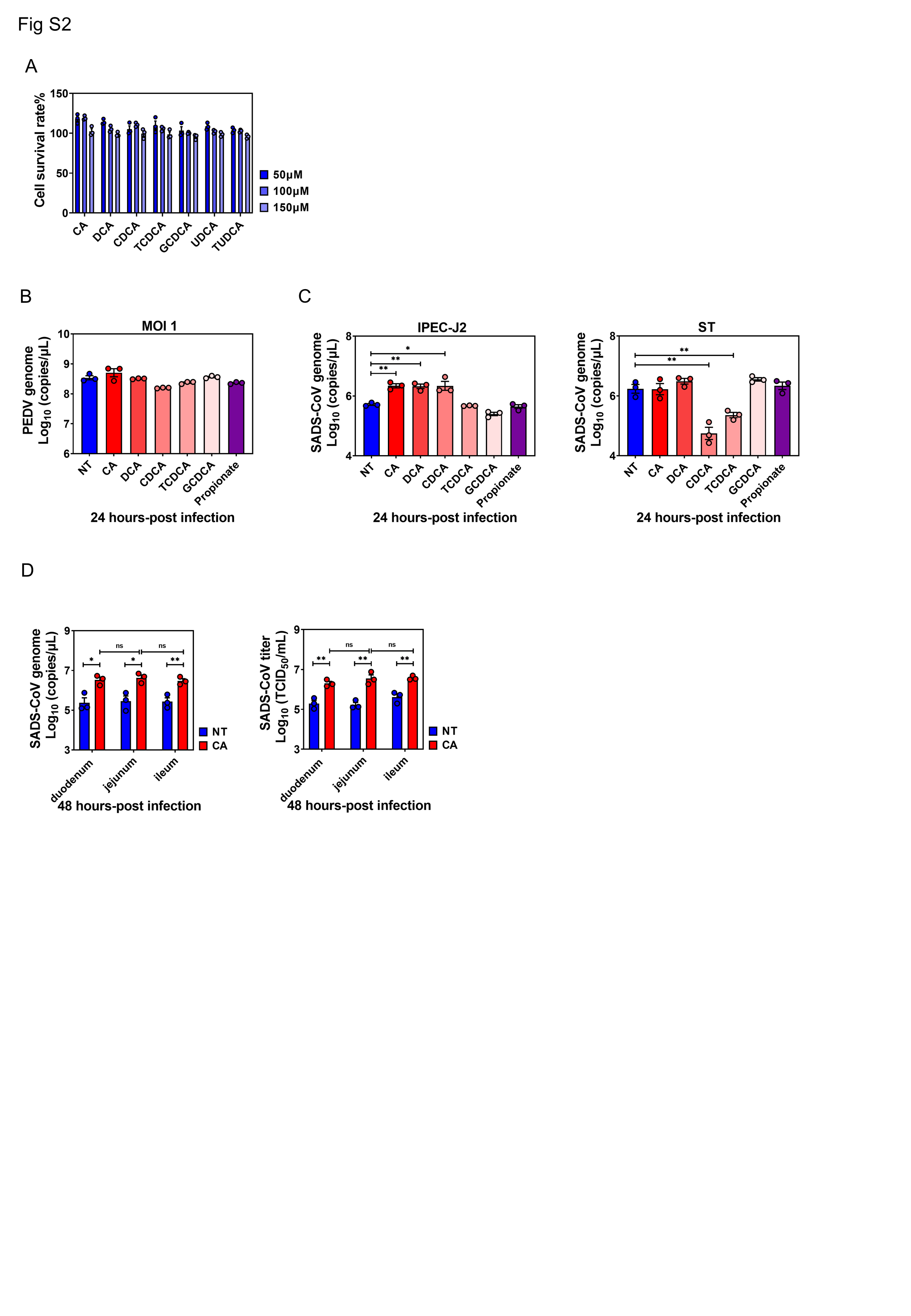

Supplement: S2 Fig — (A) Cytotoxic effect of different BAs in PIEs at the indicated concentration. (B) PIE monolayers were infected with PEDV at MOI = 1 in the presence of BAs for 24 h. Viral titer was determined by qRT-PCR. (C) IPEC-J2 and ST cells were infected with SADS-CoV-GFP at MOI = 0.1 in the presence of BAs, and viral replication was determined by qRT-PCR at 24 hpi. (D) Duodenal and jejunal PIE monolayers were infected with SADS-CoV at MOI = 0.1 in the presence or absence of cholic acid (CA) for 48 h, and viral replication was determined by qRT-PCR and TCID50 assay. Data are from three independent experiments. P values were determined by unpaired two-tailed Student’s t test. *: p < .05; **: p < .01; ***: p < .001; ns, not significant. (TIF) [file ppat.1010620.s002.TIF]

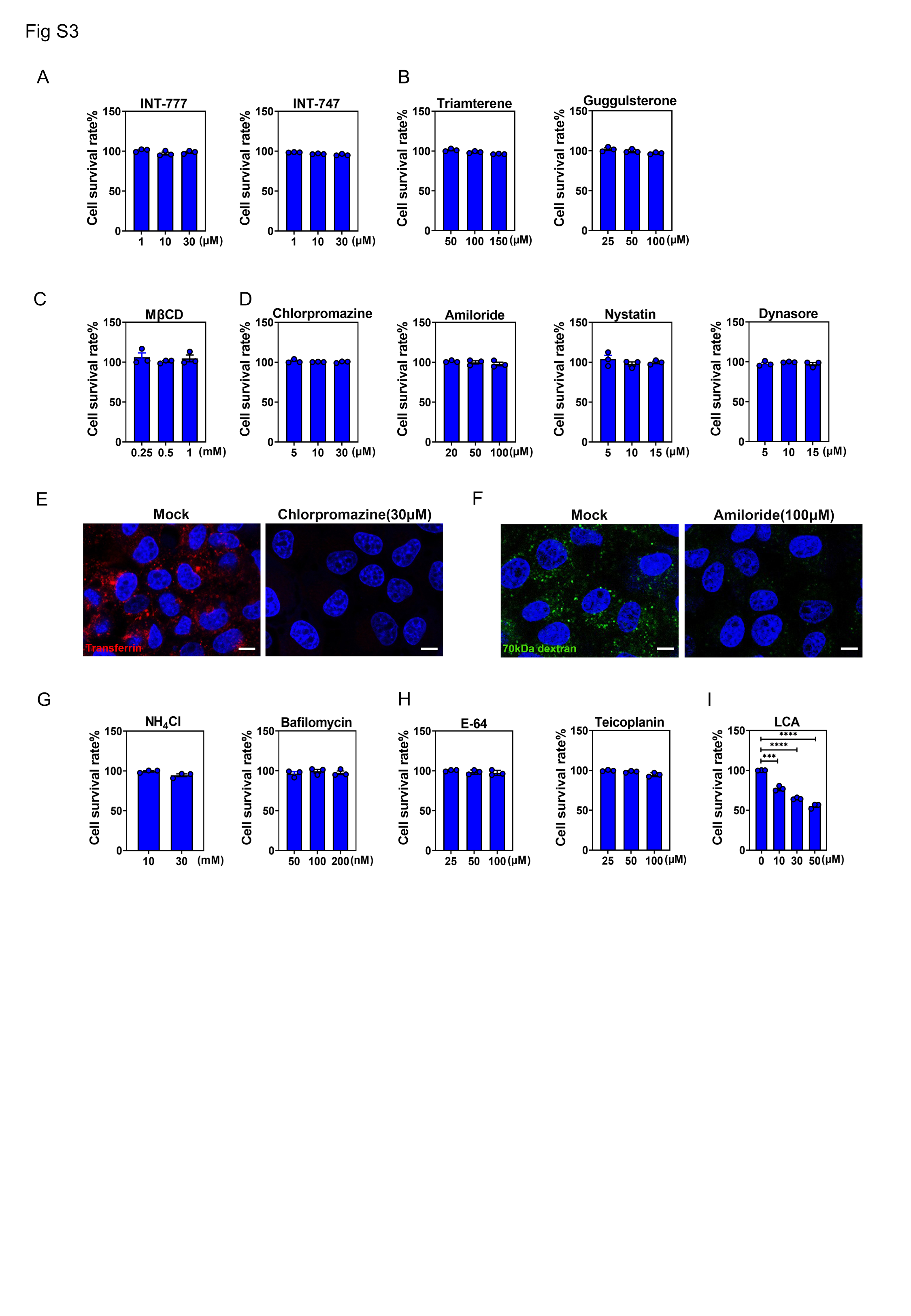

Supplement: S3 Fig — Cytotoxic effect of (A) TGR5 agonist INT-777 and FXR agonist INT-747, (B) TGR5 antagonist triamterene and FXR antagonist guggulsterone, (C) MβCD, (D) chlorpromazine (CME inhibitor), amiloride (macropinocytosis inhibitor), nystatin (CavME inhibitor) and dynasore (dynamin 2 inhibitor), in porcine intestinal enteroids (PIEs) at the indicated concentrations. (E) PIE monolayers were treated with either medium alone or 30 μM chlorpromazine for 1 h, washed with PBS three times and incubated with 25 μg/mL Alexa-594-labeled transferrin for 30 min at 37°C (scale bar, 10 μm). Images were collected on an LSM880 confocal laser-scanning microscope (Zeiss). (F) PIE monolayers were treated with either medium alone or 100 μM amiloride for 1 h, washed with PBS three times and incubated with 1mg/mL FITC-labeled 70kDa dextran for 30 min at 37°C (scale bar, 10 μm). Images were collected on an LSM880 confocal laser-scanning microscope (Zeiss). Cytotoxic effect of (G) NH4Cl and bafilomycin A1, (H) cathepsin inhibitors E-64 and teicoplanin (I) LCA in porcine intestinal enteroids (PIEs) at the indicated concentrations. (TIF) [file ppat.1010620.s003.tif]
