# Supplementary material for: Bile acids promote the caveolae-associated entry of swine acute diarrhea syndrome coronavirus in porcine intestinal enteroids
Source: PLoS Pathog. 2022 Jun 13;18(6):e1010620. doi: 10.1371/journal.ppat.1010620 (PMC9249351; doi:10.1371/journal.ppat.1010620)
Supplement: S1 Table — (DOCX) [file ppat.1010620.s004.docx]

**Primer sets used for quantitative reverse transcription-PCR**

| Gene | Sequence (5ʹ → 3ʹ) | |
| --- | --- | --- |
|  | Forward primer | Reverse primer |
| Irf3 | AAGGTTGTCCCCATGTGTCTCCG | GGAAATGTGCAGGTCCACCGTG |
| Ifnb | AGCACTGGCTGGAATGAAAC | TCCAGGATTGTCTCCAGGTC |
| Ifnl1 | CCACGTCGAACTTCAGGCTT | ATGTGCAAGTCTCCACTGGT |
| Stat1 | GCGGCAGAATTCCGACACCTGCAAC | AGCTGGCTGACGTTGGAGATCACCAC |
| Oas1 | TCCCTGGGAAGAATGTGCAG | CCCTGGCAAGAGCATAGTG |
| Isg15 | AGCATGGTCCTGTTGATGGTG | CAGAAATGGTCAGCTTGCACG |
| Mx1 | CAGAAATGGTCAGCTTGCACG | GCACTCCATCTGCAGAACTCAT |
| Ifitm1 | TGCCTCCACCGCCAAGT | GTGGCTCCGATGGTCAGAAT |
| Nlrp6 | CGGAAGGGACCAAAGAGACC | CTCCTGCGTCTCGTATAGGC |
| Caspase1 | GTGGCCATGGGTACGATCAA | GGGGCCCTTTCCGAATAACA |
| Il18 | GCTGCTGAACCGGAAGACAA | AAACACGGCTTGATGTCCCT |
| Tnfa | CGTCGCCCACGTTGTAGCCAAT | GCCCATCTGTCGGCACCACC |
| Tgr5 | AGATTAGCTGAGCGGTAGCAGG | CCATGGCTTGCCATCAAGGT |
| Fxr | TGAGCTTTGTGTCGTTTGCG | ACATTCAGCCAACATTCCCATC |
| β-actin | TGGCGCCCAGCACGATGAAG | GATGGAGGGGCCGGACTCGT |
| SADS-CoV-N | CTAAAACTAGCCCCACAGGTC | TGATTGCGAGAACGAGACTG |
| SADS-CoV-N-Probe | FAM-TGAAACCCAAACTGAGGTGTAGCAGG-TAMRA | |
| PEDV-N | CGCAAAGACTGAACCCACTAAC | TTGCCTCTGTTGTTACTTGGAGAT |
| PEDV-N-Probe | FAM-GCAGGAGTCGTGGTAATGGCAACA-TAMRA | |
